# Cell line with endogenous EGFR^vIII^ expression is a suitable model for research and drug development purposes

**DOI:** 10.18632/oncotarget.8201

**Published:** 2016-03-19

**Authors:** Wojciech J. Stec, Kamila Rosiak, Paulina Siejka, Joanna Peciak, Marta Popeda, Mateusz Banaszczyk, Roza Pawlowska, Cezary Treda, Krystyna Hulas-Bigoszewska, Sylwester Piaskowski, Ewelina Stoczynska-Fidelus, Piotr Rieske

**Affiliations:** ^1^ Research and Development Unit, Celther Polska Ltd., Lodz, Poland; ^2^ Department of Tumor Biology, Medical University of Lodz, Lodz, Poland; ^3^ Centre of Molecular and Macromolecular Studies, Polish Academy of Sciences, Lodz, Poland

**Keywords:** EGFR, EGFR^vIII^, DK-MG, glioblastoma

## Abstract

Glioblastoma is the most common and malignant brain tumor, characterized by high cellular heterogeneity. About 50% of glioblastomas are positive for *EGFR* amplification, half of which express accompanying *EGFR* mutation, encoding truncated and constitutively active receptor termed *EGFR^vIII^*. Currently, no cell models suitable for development of EGFR^vIII^-targeting drugs exist, while the available ones lack the intratumoral heterogeneity or extrachromosomal nature of EGFR^vIII^. The reports regarding the biology of EGFR^vIII^ expressed in the stable cell lines are often contradictory in observations and conclusions. In the present study, we use DK-MG cell line carrying endogenous non-modified *EGFR^vIII^* amplicons and derive a sub-line that is near depleted of amplicons, whilst remaining identical on the chromosomal level. By direct comparison of the two lines, we demonstrate positive effects of EGFR^vIII^ on cell invasiveness and populational growth as a result of elevated cell survival but not proliferation rate. Investigation of the PI3K/Akt indicated no differences between the lines, whilst NFκB pathway was over-active in the line strongly expressing EGFR^vIII^, finding further supported by the effects of NFκB pathway specific inhibitors. Taken together, these results confirm the important role of EGFR^vIII^ in intrinsic and extrinsic regulation of tumor behavior. Moreover, the proposed models are stable, making them suitable for research purposes as well as drug development process utilizing high throughput approach.

## INTRODUCTION

Glioblastoma (GB) is one of the most deadly tumors, with median survival of 12-14 months following diagnosis [1-4]. Surgical resection of GB has only limited effectiveness due to high invasiveness of tumor cells that infiltrate the surrounding brain tissue and cause disease recurrence, with inherent drug resistance of cancer cells rendering the adjuvant chemo- and radiotherapy insufficient to eliminate the invasive cells [5-8]. Novel therapies targeted at specific signaling pathways are required for effective treatment of GB [9, 10]. Approximately 50% of GBs are positive for amplification of *epidermal growth factor receptor* (*EGFR*) [11]. Overexpression of *EGFR* is accompanied in 35-60% of cases by expression of an oncogenic mutant receptor, termed variant III or vIII, that is unique to tumor tissues making it an attractive therapeutic target [12, 13]. Characterized by intragenic deletion of exons 2-7, which constitute the ligand binding domain, EGFR^vIII^ is described as constitutively active receptor. Investigation of EGFR^vIII^ expression in tumor tissue reveals a distinct pattern, with only a small portion of cells being positive for mutant receptor expression [6, 14, 15]. The effects of aberrant signaling by EGFR^vIII^ have been reported to be cell intrinsic as well as extrinsic, with a number of secreted growth factors and cytokines described [16-20]. Both autocrine as well as paracrine signaling are associated with EGFR^vIII^ expression, leading to increased cancer cell growth, survival, proliferation and altered metabolism [21-23]. Also invasiveness of cancer cells expressing EGFR^vIII^ is elevated, with positive correlation in expression of a number of metalloproteinases, MMP-9 in particular [7, 24]. Moreover, dynamic regulation of the *EGFR^vIII^* amplicon number has been reported to mediate drug resistance of glioblastoma cells [5, 25]. Taken together, those characteristics define EGFR^vIII^ as a potent oncogene and attractive therapeutic target.

At present, no therapies targeting EGFR^vIII^ are used in the clinic. One of the reasons for this is lack of appropriate models to study the biology of the receptor and, more importantly, develop novel therapeutics. Difficulties associated with establishment of EGFR^vIII^ expressing GB models are related to the loss of *EGFR* and *EGFR^vIII^* amplicons during the *in vitro* stabilization process, causes of which are unknown [26, 27]. For this reason, neurospheres from primary cancer cells or xenografts thereof are commonly used for research purposes [28]. Unfortunately, low material availability, low stability of the model (neurospheres) or high associated costs (xenografts) make those models inappropriate for drug development process, especially at the early stages of development [26, 28-30]. Alternatively, stable cell lines genetically modified to express EGFR^vIII^ are used [31], however, such models do not account for tumor tissue heterogeneity or extrachromosomal nature of *EGFR^vIII^*. Furthermore, reports on direct activation of the signaling pathways, trans-activation of other receptor tyrosine kinases and secretome alterations resulting from EGFR^vIII^ expression vary greatly (e.g. Erdem-Eraslan et al., 2015 [32] and Puliyappadamba et al., 2013 [33]). This demonstrates the need for an appropriate cellular model that depicts the nature of glioblastoma, has extrachromosomal *EGFR^vIII^* and is suitable for high throughput studies utilized in drug development.

## RESULTS

### Analysis of currently used *in vitro* glioblastoma models

**Figure 1:**
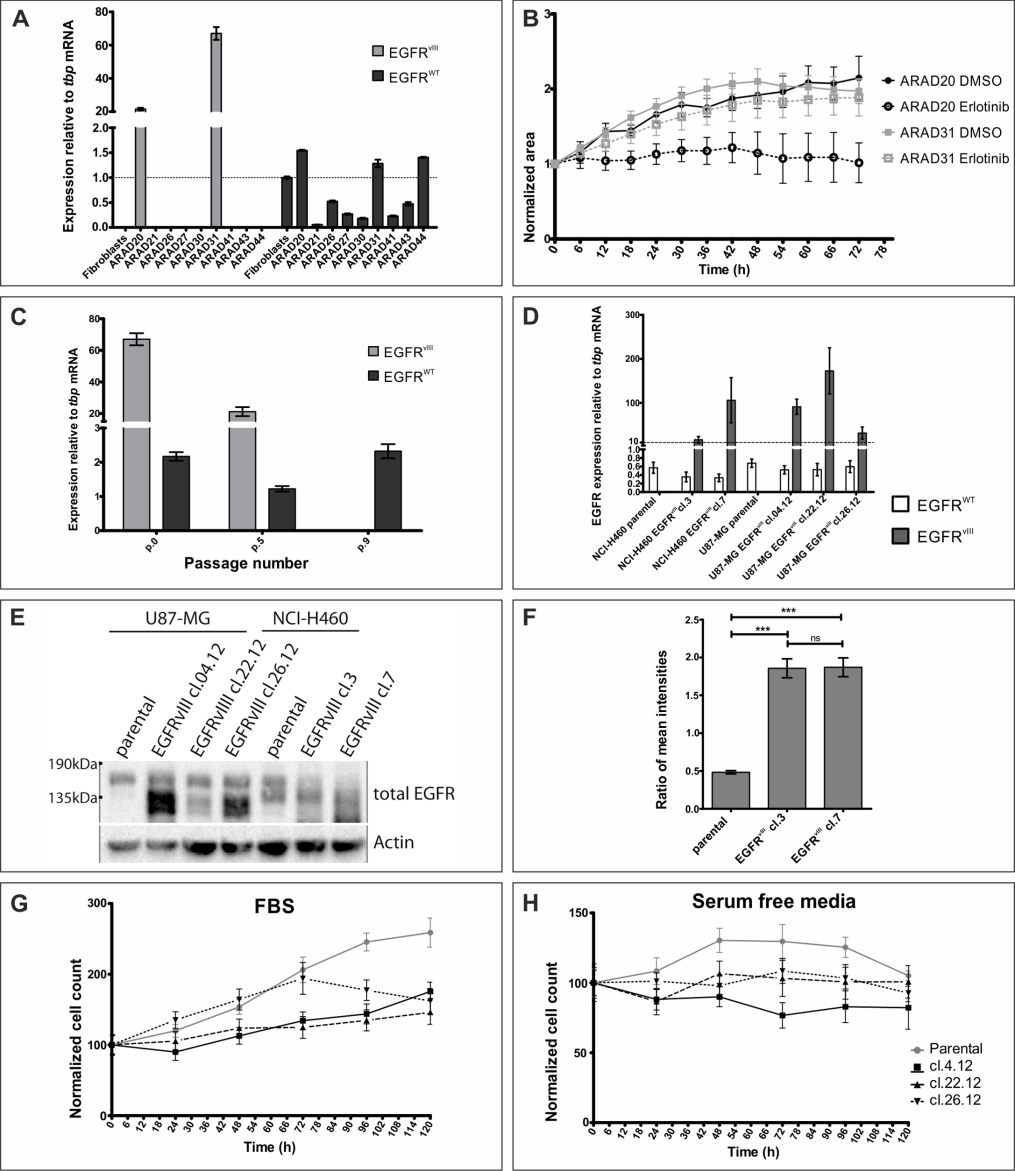
Assessment of models currently used to study EGFR^vIII^ **A.** Glioblastoma samples were analyzed on the mRNA level for EGFR^vIII^ and EGFR^WT^ expression. **B.** Neurospheres obtained from glioblastoma resections positive for EGFR^vIII^ expression were treated with DMSO or erlotinib (10 μM). At least 3 neurospheres were analysed in each condition. **C.** Adherent cell line established from ARAD31 was cultured over several passages and *EGFR*^vIII^ and *EGFR*^WT^ mRNA levels were monitored. **D.** Stable cell lines, U87-MG and NCI-H460, were modified to express EGFR^vIII^. Graph shows mRNA levels of EGFR^WT^ and EGFR^vIII^. **E.** Western blot analysis of same cell lines as in D. Expected size: EGFR^WT^ - 175kDa, EGFR^vIII^ - 135kDa. Numbers to the left indicate molecular size. Arrows indicate not specific bands in NCI-H460 line. **F.** Ratio of an average mean fluorescence signal intensity per cell for total EGFR and EGFR^WT^, an approximation of EGFR^vIII^ expression, is shown for NCI-H460 cell lines. (G and H) Populational growth of U87-MG cell lines in culturing medium supplemented with 10% FBS **G.** or without any supplements **H.** is shown. Legend to both graphs is on the right-hand side of H. Error bars indicate SEM. Statistical significance determined by ANOVA analysis with post-analysis Bonferroni's multiple comparisons test. ***, *p* < 0.05; ns, not significant.

Investigation of the protein activity is best conducted in the environment as close to the native as possible, allowing for insight into the functional biology of the protein. Therefore, we have attempted using neurospheres formed by primary cell cultures obtained from surgical resections. Despite problems with stabilization of the primary cell cultures reported previously [27], we have analyzed nine glioblastoma resections, two of which were positive for EGFR^vIII^ transcript (Figure 1A). Treatment of EGFR^vIII^-positive neurospheres with erlotinib produced variable results between tumors (Figure 1B and Sup.Figure 1A). Analogous situation was observed upon treatment with EGF, with 50% of spheres from the same tumor not showing any effect and the remaining ones displaying signs of cell death (Sup.Figure 1B). Our attempts at stabilization of the primary glioblastoma cells positive for EGFR^vIII^ in the form of an adherent cell line was only partially successful for only one of the tumors, with cancer cells surviving post-passage 10 without *EGFR^vIII^* amplicons. RT-PCR analysis of the EGFR^vIII^ mRNA levels clearly indicated a rapid decline (Figure 1C), consistent with reports in the literature [26, 27].

**Figure 2:**
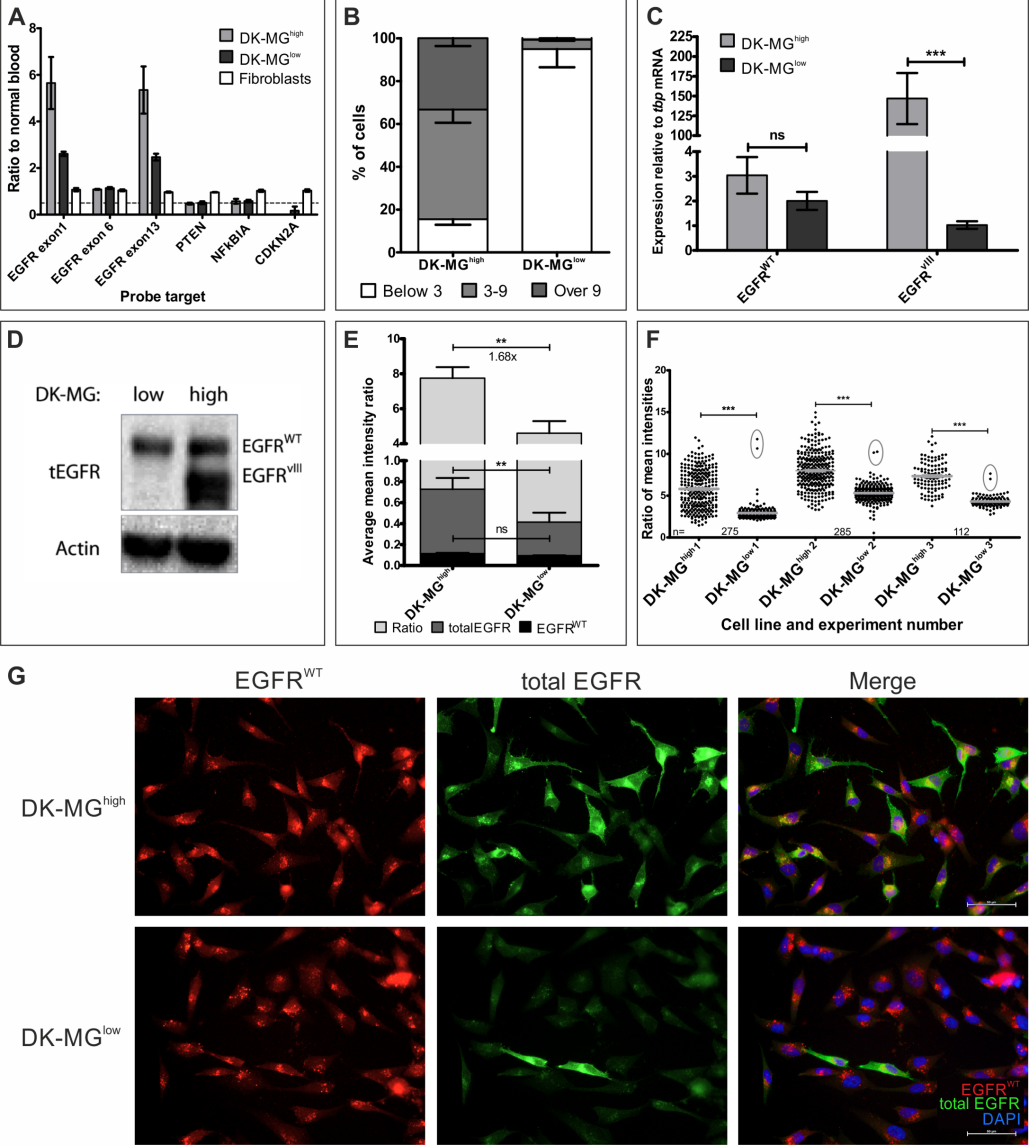
Characterization of the DK-MG^low^ and DK-MG^high^ cell lines **A.** MLPA analysis of the DK-MG lines in comparison to fibroblasts. Dashed line marks ratio of 0.5. **B.** Quantification of the composition of two DK-MG lines with regards to the number of amplicons per nuclei. **C.** Investigation of *EGFR*^WT^ and *EGFR*^vIII^ mRNA levels in DK-MG lines. **D.** Western blot comparison of both DK-MG lines using antibody recognizing wild-type and mutant EGFR. **E.** Quantification of an average mean fluorescence signal intensity per cell for total EGFR, EGFR^WT^ and total EGFR/EGFR^WT^ ratio, an approximation of EGFR^vIII^ expression, is shown. Fold change of ratio is shown below the top statistical bar. **F.** Scatter plot of antibody signal intensity ratio for individual cells in each experiment summarized in E is shown. Cells with very high ratio in DK-MG^low^ line are circled. Grey lines indicate average value with SEM, n for each line in experiment is given. **G.** Representative images of EGF-mediated degradation of EGFR^WT^, observed as intracellular dots, following 1 h treatment with EGF. Cells strongly positive for total EGFR are likely to be EGFR^vIII^ positive. Images obtained using 20x objective. Error bars indicate SEM. Statistical significance calculated by Student's *t*-test. *, *p* < 0.05; **, *p* < 0.01; ***, *p* < 0.005; ns, not significant.

With stable cell lines offering a less variable model, we attempted inserting *EGFR^vIII^* cDNA under the control of the constitutively active CMV promoter into U87-MG and NCI-H460 cell lines using lipofection or lentiviral transduction, respectively. A couple of stable clones were established from both cell lines, however, expression of the transgene varied among them on the mRNA level, despite the same transfection protocol (Figure 1D). Assessment of EGFR^vIII^ expression on the protein level in H460 line using western blotting proved impossible, as a non-specific band was present around 135kDa, size expected for EGFR^vIII^ (Figure 1E). To assure that protein is synthesized and delivered to the correct subcellular localization, we performed immunofluorescent staining (Figure 1F and Sup.Figure 2). Reportedly low specificity of commercially available antibodies against EGFR^vIII^ prompted us to evaluate expression of EGFR^vIII^ as a ratio of total EGFR (antibody recognizes intracellular domain, Sup.Figure 2A) and wild-type receptor (antibody binds to the domain missing from EGFR^vIII^, Sup.Figure 2B) [6]. Such approach did confirm the presence of EGFR^vIII^ protein in transduced cells, which displayed a level of heterogeneity despite antibiotic selection (Sup.Figure 2C), however, no correlation between mRNA and protein levels was observed. Furthermore, genetic manipulation had an effect on EGFR^WT^ levels with immunofluorescent staining and western blot data indicating decreased levels in both transduced clones in comparison to the parental line (Sup.Figure 2B and Figure 1E, respectively). For those reasons, we did not continue our study with the NCI-H460 line.

**Figure 3:**
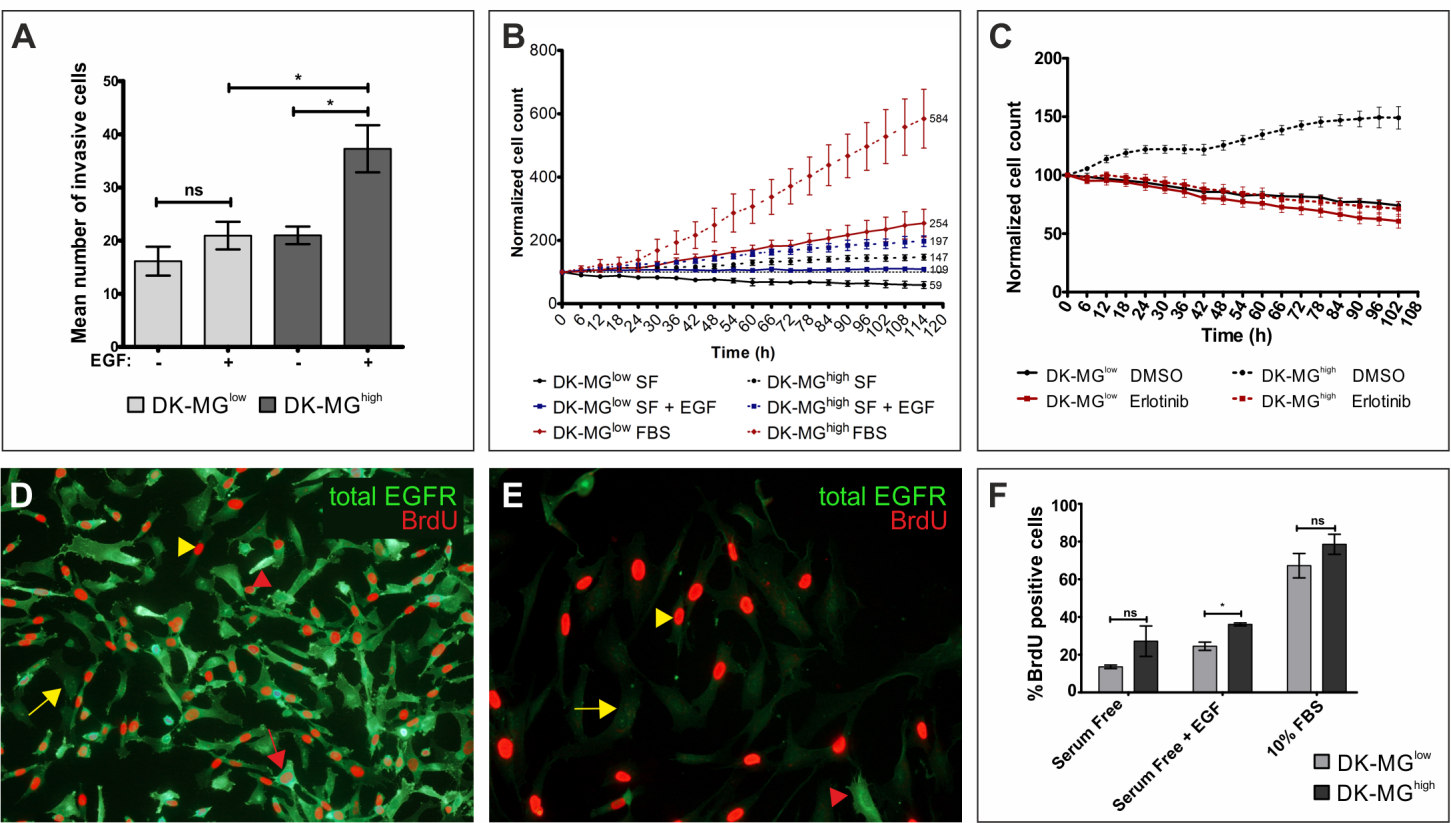
Relationship between expression of EGFR^vIII^ and the biology of cell population **A.** Invasiveness of DK-MG cell lines was measured on matrigel invasion transwells three days after the initial seeding, with EGF serving as an attractant. **B.** Populational growth of both cell lines, DK-MG^low^ (continuous line) and DK-MG^high^ (dashed line), in serum free media (black circle) supplemented with 20 ng/mL EGF (blue square) or 10% FBS (red diamonds) is shown as a percentage of initial number of cells. Numbers on the right side of the plot indicate the final cell count. **C.** Populational growth rate for both DK-MG lines upon treatment with 10 μM erlotinib or control DMSO. (D and E) Representative image depicting BrdU incorporation into DK-MG^high^ **D.** and DK-MG^low^ **E.** cell line after 5 days in serum free media. Red arrow: high total EGFR and BrdU-positive, red arrowhead: high EGFR and BrdU-negative, yellow arrow: low EGFR and BrdU-positive, yellow arrow: low EGFR and BrdU-negative. 20x and 40x objectives were used, respectively in D and E. **F.** Quantification of BrdU incorporation expressed as a percentage of BrdU positive cells in the population after 1 day of cell culture under the indicated culture conditions. Error bars indicate SEM. Statistical significance calculated by paired Student's *t*-test. *, *p* < 0.05; ns, not significant.

U87-MG is a line commonly used to study the biology of EGFR^vIII^ [31]. Lipofection of U87-MG line produced three clones expressing EGFR^vIII^ on the mRNA and protein level, although no correlation between the RT-PCR results (Figure 1D) and western blot analysis (Figure 1E) was observed. This is particularly true for clone 22.12, which had the highest mRNA levels and markedly lowest protein expression of the three clones (Figure 1E). To investigate the effect of EGFR^vIII^ expression on the biology of cell population, we have monitored the number of cells over a five day period (Figure 1G and 1H and Sup.Figure 3). Surprisingly, introduction of EGFR^vIII^ into U87-MG line decreased the proliferative potential of the line in complete medium (Figure 1G). Furthermore, resistance to stress, depicted here as serum starvation, was not changed following expression of EGFR^vIII^ (U87-MG). Finally, negative effect of EGF stimulation on cell viability was previously reported for this model [33]. Surprisingly, we did not observe any changes in cell number upon culturing U87-MG lines in medium supplemented with EGF, despite presence of the wild-type receptor of the ligand (Sup.Figure 3). This might suggest lack of machinery necessary to transduce EGFR^vIII^ signaling or mutations downstream of EGFR^vIII^ that initiate receptor-independent signaling, undermining reliability of the model. Variability in mRNA and protein levels observed despite the same lipofection protocol, as well as discrepancies between reported and obtained results formed the basis for discontinuing studies using genetically modified lines.

### Characterization of DK-MG lines

**Figure 4:**
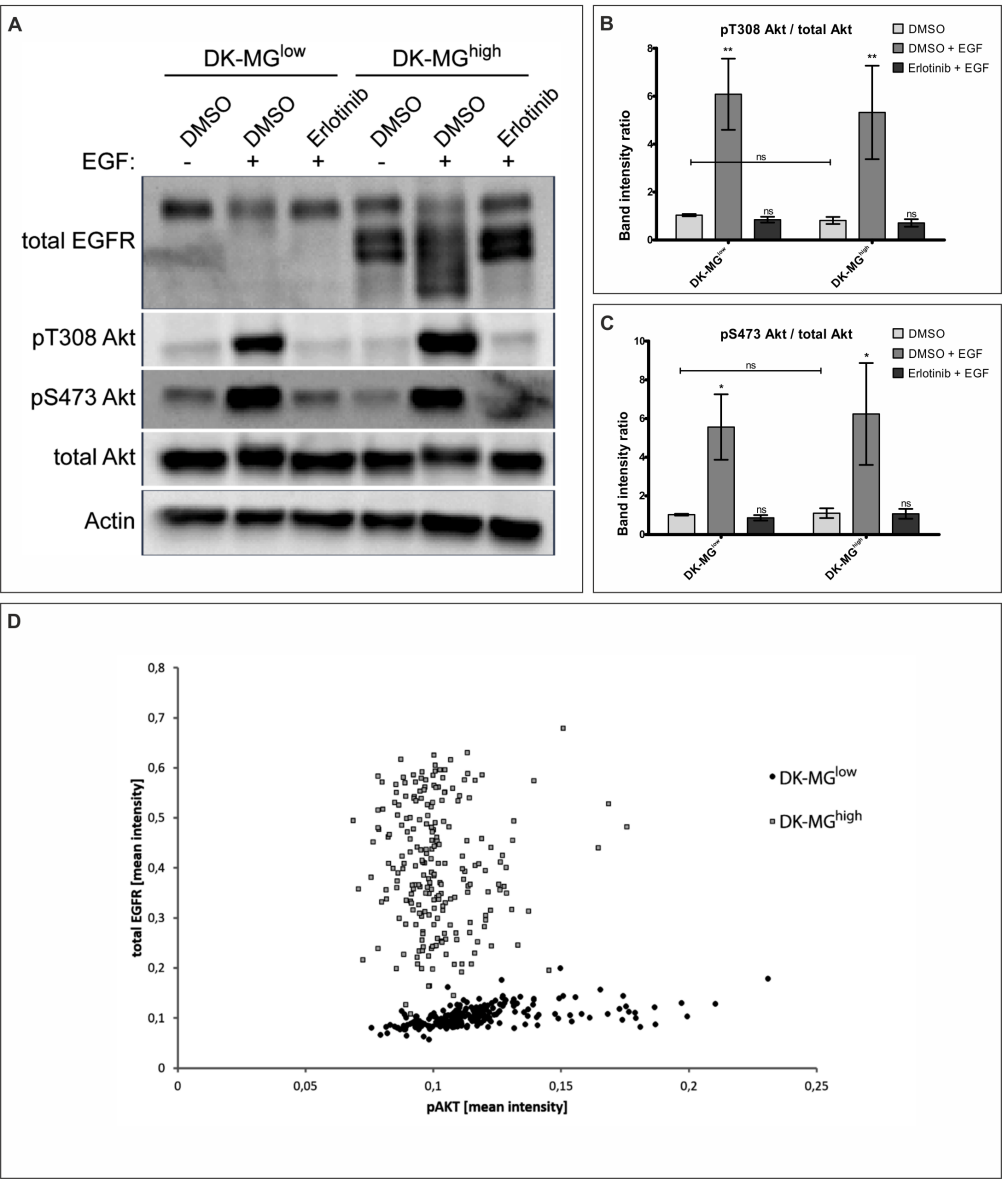
DK-MG cell lines do not differ in PI3K/Akt signaling pathway activation levels **A.** DK-MG^low^ and DK-MG^high^ lines pre-treated with DMSO or erlotinib were stimulated with EGF as indicated prior to lysis and analysis *via* western blot. Representative image is shown. Phosphorylation of Akt on Threonine 308 and Serine 473 was assessed with specific antibodies. (B and C) Quantification of blot in A, displaying ratio of band density of pT308 **B.** and pS473 **C.** over total Akt is shown. Asterisks over bars indicate statistical significance towards DMSO control for the line tested. **D.** Scatter plot indicating correlation between mean intensity of antibodies against total EGFR (Y-axis) and pS473 Akt (X-axis) per cell is shown. Each dot represents individual cell, with black dots representing DK-MG^low^ and grey squares representing DK-MG^high^ cells.Error bars indicate SEM. Statistical significance calculated by two-way ANOVA with Bonferroni's post-comparison test; ***, *p* < 0.005; **, *p* < 0.01; *, *p* < 0.05; ns, not statistically significant.

**Figure 5:**
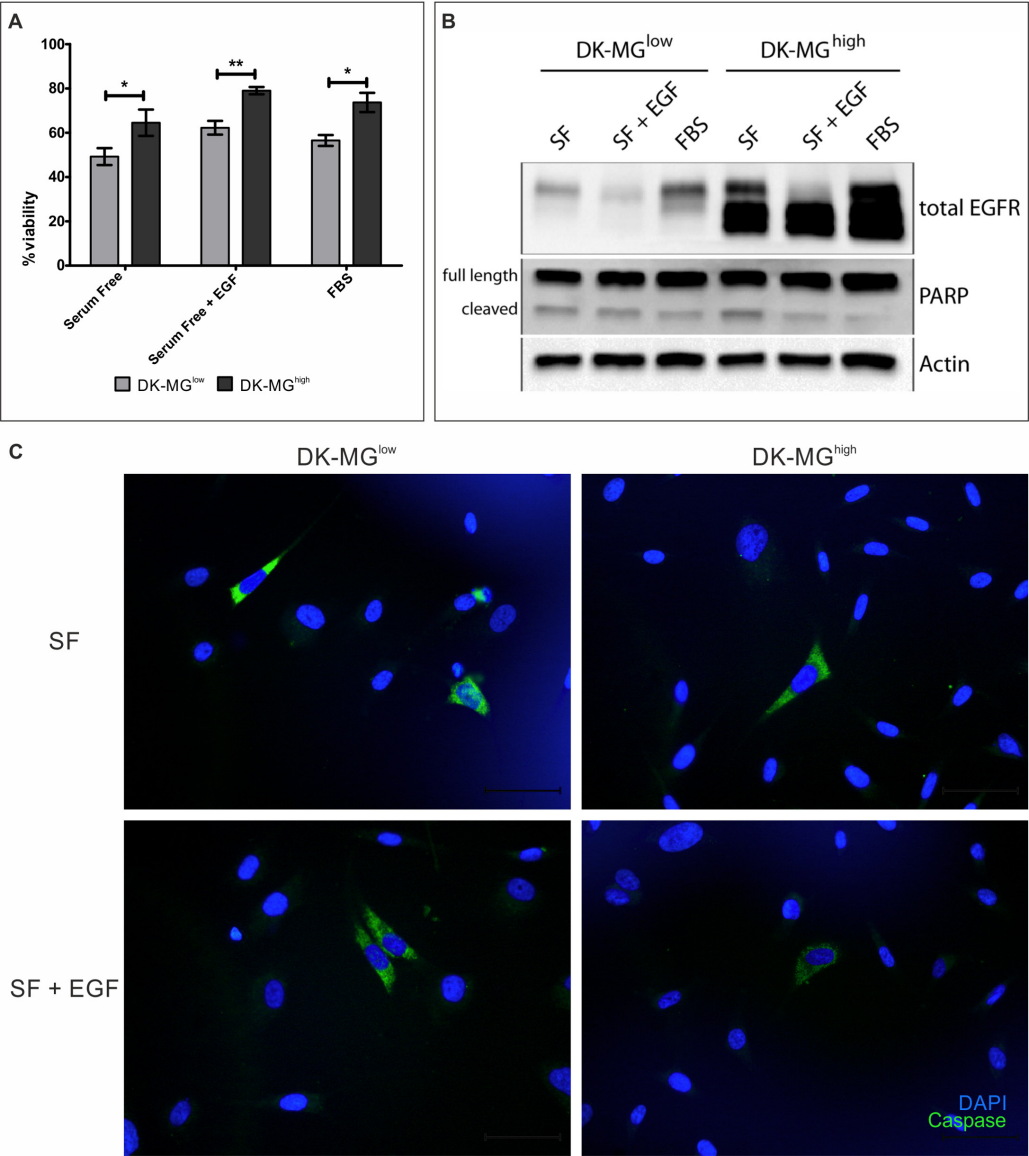
EGFR^vIII^ affects viability of DK-MG cell lines **A.** Comparison of viability of both DK-MG cell lines, measured by propidium iodide incorporation after 3 days of culturing under the indicated conditions. **B.** Cleavage of PARP for both cell lines was assessed using western blotting. Full length and cleaved forms of PARP are indicated on the left hand side of panels. **C.** Representative images showing activity of the synthetic Caspase 3/7 reporter in serum free media (top panel) or supplemented with 20 ng/mL EGF (bottom panel). The number of reporter positive cells was higher in the DK-MG^low^ line under both culture conditions, without any signs of clumping of apoptotic cells. 40x objective used. Error bars indicate SEM. Statistical significance calculated by paired Student's *t*-test. *, *p* < 0.05; **, *p* < 0.01; ***, *p* < 0.005.

To study the biology of the mutant EGF receptor we utilized cell line previously reported to express EGFR^vIII^ endogenously. Del Vecchio and colleagues have characterized DK-MG cells as heterogenous line with populations positive and negative for EGFR^vIII^ expression [13]. We have attempted establishing cell line that is free of *EGFR^vIII^* amplicons. Due to the reported low specificity of the anti-EGFRvIII antibodies as well as an unknown effect of those antibodies on cell biology, we have chosen not to use cell sorting, but rather, a serial dilution method. Interestingly, despite establishing numerous clones with varying levels of EGFR^vIII^ expression, we were unable to stabilize a single cell line completely free of EGFR^vIII^ amplicons. For the purpose of this study, we have used clones with the lowest and high levels of EGFR^vIII^, referred to as DK-MG^low^ and DK-MG^high^, respectively. MLPA analysis did not reveal any differences between DK-MG lines, other than EGFR^vIII^, with both lines having normal number of wild-type *EGFR* copies, hemizygous *PTEN* and *NFkBIA,* and *CDKN2A* deleted; all mutations reminiscent of brain tumors (Figure 2A) [5, 34, 35]. The differences in *EGFR^vIII^* DNA levels can be explained by, either, a decrease in the number of cells positive for amplicons or a decrease in the average number of amplicons per cell. FISH analysis indicated that cells without EGFR^vIII^ amplicons constituted much higher fraction of the overall DK-MG^low^ population, compared to the DK-MG^high^ line (Figure 2B and Sup.Figure 4). Only 5% of DK-MG^low^ population had more than 2 amplicons per cell, in contrast to DK-MG^high^ line that had 85% of cells positive for multiple amplicons. Furthermore, FISH analysis confirmed the extrachromosomal nature of EGFR^vIII^ amplicons (Sup.Figure 5) [14, 25].

The drastic difference in the number of cells positive for EGFR^vIII^ amplicons was reflected in the overall EGFR^vIII^ mRNA and protein levels, with DK-MG^high^ line being more abundant in the mutant receptor's transcript and protein (Figure 2C and 2D, respectively). To investigate protein expression on individual cell basis, we utilized immunofluorescent staining technique that we used for assessment of NCI-H460 cell line (Figure 2E). Results of western blot analysis as well as quantification of the fluorescence intensity indicated that the amount of EGFR^WT^ in DK-MG^high^ and DK-MG^low^ lines was very similar, in contrast to the amount of total EGFR per cell that was much higher in DK-MG^high^ cell line, indicating that EGFR^vIII^ must be the differentiating factor (Figure 2D and 2E). Approximately 1% of DK-MG^low^ cells had strongly elevated ratio, implying expression of EGFR^vIII^ (Figure 2F). An alternative approach relied on the phenomenon of EGF-mediated endocytosis of the wild-type receptor, which should leave only stable EGFR^vIII^ in the plasma membrane [36-38]. Following stimulation with EGF, the majority of cells in the DK-MG^low^ line had signal emanating from small vesicular intracellular compartments, reminiscent of endosomes, with occasional cells staining uniformly throughout the cell body (Figure 2G). Significantly more cells (over 50%) remained uniformly stained in the DK-MG^high^ line following similar treatment, however no distinctive populations could be detected due to high heterogeneity of the line (Figure 2F and 2G).

The ability to compare two cell lines with highly similar genetic makeup, yet differing in the EGFR^vIII^ expression, presents a unique opportunity to study the role of EGFR^vIII^ in cell biology, especially in the context of cell population. However, to exclude the possibility that genetic alteration other than *EGFR^vIII^* levels has impact on cell and cell population biology, we have performed next generation sequencing of a panel of 408 genes commonly associated with cancer, using Comprehensive Cancer Panel on Ion Torrent PGM (Sup.Table.1). Detailed analysis revealed presence of a number of mutations, however, no major discrepancy in the mutation profile between two lines was detected.

**Figure 6:**
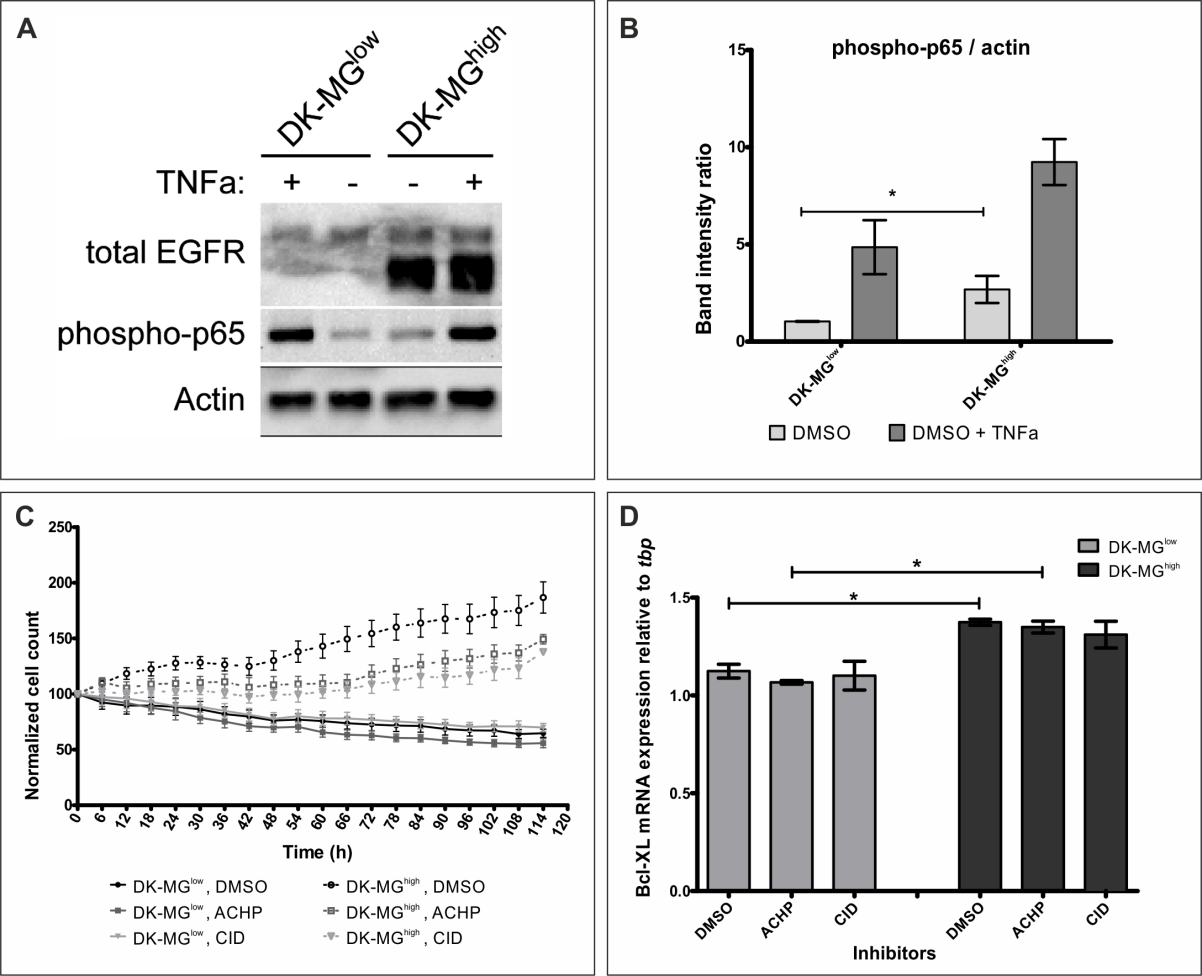
EGFR^vIII^ aberrantly activates the NFκB pathway **A.** Representative western blot analysis of the DK-MG lines treated for 20min with TNF, as indicated. **B.** Quantification of 6 independent western blots as shown in A. phosphorylated p65 band was normalized to Actin for quantification purposes. Under steady state conditions NFκB pathway is active to higher degree in DK-MG^high^ line compared to the DK-MG^low^ line. **C.** Size of the DK-MG^low^ (continuous line) and DK-MG^high^ (dashed line) populations over time when cultured in serum free media with DMSO (black dot), ACHP at 2.5μM (grey square) or CID2858522 at 1μM (light grey diamond) added. Both NFκB inhibitors affect populational growth of the DK-MG^high^ line, but not the DK-MG^low^ line. The number of cells is statistically different for DK-MG^high^ line between DMSO treated and ACHP and CID treated from 48h and 60h, respectively (as determined by two-way ANOVA with Bonferroni's post-test, *p* < 0.05). **D.** mRNA levels of *Bcl-XL* was measured in DK-MG cell lines incubated with NFκB pathway inhibitors or control carrier for 3 days. Inhibitors did not affect *Bcl-XL* expression in either cell line, however, difference between the two lines can be observed. Error bars indicate SEM. Statistical significance calculated by paired Student's *t*-test. *, *p* < 0.05; **, *p* < 0.01; ***, *p* < 0.005; ns, not significant.

Importantly, numerous reports indicated rapid decrease in the number of extracellular amplicons during any attempt of culturing glioblastoma cells *in vitro* [26]. We have evaluated the EGFR^vIII^ transcript levels as well as amplicon number over an extensive number of passages (Sup.Figure 6A and 6B, respectively). We did not detect any significant differences between cells in early and late passages, indicating stability of the genetic makeup of the cellular population.

### Function of EGFR^vIII^ in the context of cell population

One of the major hallmarks of tumor cells is their ability to penetrate the extracellular matrix. Study of cell invasiveness using Matrigel trans-wells indicated statistically significant difference between cells cultured with and without attractant presence (Figure 3A). Consistent with the previous reports indicating that EGF can act as an attractant during chemotactic invasion, we have observed that addition of EGF to the bottom side of the trans-wells resulted in a significant increase in the number of invading cells in the DK-MG^high^ line, but not in the DK-MG^low^ line [39]. This implies that EGFR^vIII^ contributes towards elevated invasiveness of cells, even though it cannot bind the ligand itself. The golden standard experiment in cancerogenesis is the ability of cells to form tumors *in vivo* [28]. We have attempted inoculating immunocompromised mice (SCID Hairless Outbred, SHO line) with both DK-MG cell lines and measured tumor volume over a course of 6 weeks (Table 1). To make a direct comparison of DK-MG lines, each mouse was inoculated with DK-MG^low^ and DK-MG^high^ cells injected into opposite flanks of the animal. Half of animals did not present with tumor masses on flanks injected with DK-MG^low^ line, in contrast to DK-MG^high^ cells that produced solid masses in all mice. Additionally, one mouse displayed regression of the DK-MG^low^ cells-induced tumor mass. Despite significant differences in tumor growth and size, these data confirms that EGFR^vIII^ contributes towards invasiveness of cancer cells.

**Table 1:** Mice inoculated with DK-MG cells

Mouse	Matrigel		Day (mm^3^)		
		Inoculation	14	21	40
1	Full	DK-MG^high^	26.51	26.70	18.38
		DK-MG^low^	none	none	none
2	GFR	DK-MG^high^	15.48	16.78	21.39
		DK-MG^low^	48.44	57.81	62.06
3	Full	DK-MG^high^	27.78	71.84	58.16
		DK-MG^low^	27.26	11.59	none
4	GFR	DK-MG^high^	16.34	47.52	56.27
		DK-MG^low^	none	none	none

SHO mice were inoculated with a mixture of 2×10^5^DK-MG cells with matrigel. DK-MG^low^ cells were injected subcutaneously into the right flank of the animal, DK-MG^high^ cells were injected into the left flank of the animal. Full or growth factor reduced (GFR) matrigel was used in the experiment. Table shows the volume (in cubic mm) of solid tumors formed.

Another significant difference between two cell lines that confirms EGFR^vIII^ to be an oncogene is the rate of populational growth (Figure 3B). DK-MG^high^ line grew at a faster pace than the DK-MG^low^ line under the same culture conditions. Interestingly, under serum-free conditions, the EGFR^vIII^-higly expressing line did proliferate at a moderate rate, in contrast to the EGFR^vIII^-low line that showed a decrease in cell number. Addition of EGF rescued DK-MG^low^ line from reduction of the cell number and elevated growth of DK-MG^high^ line. This implies that EGF acts on DK-MG cells not only as an attractant, but also as a growth factor. Moreover, aberrant EGFR^vIII^ signaling does not saturate the signal transduction machinery of the cell, confirming the qualitative difference between the wild-type and mutant receptors, suggested previously [33, 40]. To confirm that elevated growth rate of DK-MG^high^ line is a result of deregulated EGFR signaling pathway, we used an inhibitor of the kinase domain of EGFR, erlotinib, which does not discriminate wild-type EGFR from its mutant version (Figure 3C). Erlotinib did not affect populational growth of the DK-MG^low^ line, however it did negate the positive effect of EGFR^vIII^ on the population growth in the DK-MG^high^ line.

As the DK-MG^high^ population is very heterogenous for EGFR expression, we have attempted investigating whether the amount of total EGFR in cells correlates with the proliferation rate, measured by BrdU incorporation. Surprisingly, we observed that cells strongly positive for EGFR^vIII^ as well as those deprived of it, within the same population, undergo genome replication at an equal rate (Figure 3D). Under the same conditions, cells in the DK-MG^low^ line negative for EGFR^vIII^ expression replicate DNA very effectively (Figure 3E). When we compared BrdU incorporation in DK-MG^high^ and DK-MG^low^ line, the former line displayed higher incorporation rate when cultured in serum free and serum free with EGF supplemented conditions, albeit statistical significance was reached only in the presence of EGF (Figure 3F). Culturing of cells in complete medium (10% FBS) did not produce any differences between cell lines.

### PI3K/Akt signaling pathway is activated to the same extent in both DK-MG cell lines

Considering that canonical EGFR signaling activates PI3K/Akt pathway, which is associated with regulation of cell proliferation, we have investigated phosphorylation of Akt (Figure 4A, quantified in B and C). As Akt can be activated by phosphorylation of two independent residues, Serine 473 and Threonine 308, we have assessed both sites for post-translational modification. Both residues did undergo change in the phosphorylation status following stimulation with EGF, as expected. Comparison of the phosphorylation level under steady-state conditions did not reveal any differences between DK-MG^high^ and DK-MG^low^ lines (Figure 4A-4C). In order to assess the direct correlation between abundance of EGFRvIII and Akt phosphorylation, we have performed co-staining with antibodies recognizing total EGFR as well as phosphorylated Akt on S473 (Figure 4D). No correlation between the amount of EGFR^vIII^ (extrapolated from the constant level of EGFR^WT^ between cells) and Akt phosphorylation was observed.

### EGFR^vIII^ contributes towards elevated viability of cells

Considering the differences between cell lines in the rate of population growth (Figure 3B) and very limited difference in DNA replication rate (Figure 3E), it is possible that cells in the DK-MG^low^ population undergo cell death more often than cells in the EGFR^vIII^- highly expressing population do. To test this hypothesis, we have investigated propidium iodide incorporation as a measure of cell viability (Figure 5A). As expected, DK-MG^low^ cells displayed lower viability than their counterparts, under all culture conditions. Interestingly, similar portion of population of each cell line underwent cell death, irrespective of culture conditions. To elucidate the mechanism of cell death, we have performed a western blot analysis looking at cleavage of PARP, a substrate of the Caspase family of proteases. More cleaved form of PARP was observed in the DK-MG^low^ line in cells cultured in serum free medium supplemented with EGF as well as 10% FBS (Figure 5B). Interestingly, cells cultured in serum free media without any supplements had similar levels of cleaved PARP between lines. Confirmation of this result came from the synthetic reporter of Caspase 3/7 activity, which stained more cells in the DK-MG^low^ line compared to the DK-MG^high^ line (Figure 5C). Both of those results implicate apoptosis as a means of cell death, which occurs more often in the DK-MG^low^ line.

### Elevated EGFR^vIII^ expression correlates with NFκB signaling pathway activation

One of the main signaling pathways playing a pro-survival role is the Nuclear Factor κ B (NFκB) signaling pathway, which has been previously shown to be affected by the EGFRvIII signaling [40-42]. To assess whether this is the molecular mechanism underlying the differences in cell viability between the two DK-MG cell lines, we have assessed phosphorylation of p65, as a measure of pathway activation (Figure 6A, quantified in 6B). In both cell lines, treatment with TNFα, a ligand described to result in NFκB pathway activation, caused a strong increase in p65 phosphorylation. Comparison of the two DK-MG cell lines indicated stronger activity of the NFκB pathway in the DK-MG^high^ line under steady-state, serum-free conditions. Confirmation of the NFκB pathway playing a differentiating role between the two DK-MG cell lines came from populational growth study performed in the presence of inhibitors CID2858522 and ACHP (Figure 6C). Importantly, only DK-MG^high^ line was affected by the inhibitors, significantly decreasing the populational growth rate, but not to the level observed in the DK-MG^low^ line. Indicated inhibitors were able to decrease the p65 phosphorylation only in the DK-MG^high^ line (Sup.Figure 7).

Interestingly, previous reports indicated a relationship between expression of EGFR^vIII^, activity of the NFκB pathway and expression of the members of Bcl family of pro-survival proteins, including Bcl-XL [43, 44]. Investigation of the mRNA levels of the *BCL-XL* gene indicated that DK-MG^high^ cells had higher levels of transcript under steady-state conditions, which would correlate with elevated viability of the line (Figure 6D) [45]. However, treatment with NFκB pathway inhibitors, ACHP or CID2858522, has not decreased the mRNA levels of Bcl-XL. Taken together, these data indicate that EGFR^vIII^ is involved in protecting cells from apoptosis, however the molecular mechanism is not linear *via* NFκB pathway and remains to be elucidated.

## DISCUSSION

Despite EGFR^vIII^ being an attractive therapeutic target, no treatments focusing on it are used in the clinic [10, 46]. One of the major obstacles in development of effective drugs for treatment of glioblastoma is lack of relevant models, suitable for early-stage drug development studies often requiring high-throughput approaches. Majority of studies are conducted on cell lines ectopically expressing the mutant receptor or neurospheres [6, 26, 47]. Our experience indicates that neurospheres are not suitable for high throughput studies due to limited material availability, experimental variability and issues with stability of model's molecular profile (Figure 1A-1C and Sup.Figure 1). Those results reflect findings reported in the literature on the loss of *EGFR^vIII^* and *EGFR^WT^* amplicons [26, 29, 48-51]. The neurospheres made of glioblastoma cells proved difficult to maintain over prolonged periods of time (more than 15 passages), with senescence described as the predominant cause, irrespective of the culture conditions [30, 52, 53]. Fact that spheroids can not to be cultured infinitely *in vitro* forced Johnson and colleagues to use immunocompromised mice as neurosphere “incubators,” with serial xenografting of the neurospheres between animals [28]. This approach is not viable for all purposes due to bioethical issues, high costs associated with the use of murine models and relatively difficult access to animal facilities suitable for maintaining SCID mice. Finally, the uniqueness of the genetic profile of the patient naturally introduces high degree of variability into the model, questioning the reproducibility of data. With that in mind, it is reasonable to use patient-derived neurospheres to confirm the key findings or to validate the efficiency of the developed therapeutic strategy. On the other hand, genetically engineered stable cell lines can vary between laboratories depending on their preparation methodologies and culture conditions, as evidenced by our own experience and contradicting results (i.e. effects of EGF stimulation on cells) reported in the literature [32, 33] (Figure 1D-1G and Sup.Figure 2). Furthermore, such models do not take into account the extrachromosomal nature of EGFR^vIII^, which has an impact on drug resistance and can be a significant obstacle in development of novel therapeutics [25, 46]. The proposed DK-MG cellular models are therefore highly attractive tools to study the biology of EGFR^vIII^ as well as to develop drugs targeting the oncogenic receptor, as all of the aforementioned aspects are encompassed by the proposed model - reproducibility, limited costs and reflection of the tumor biology.

Clonal selection of a cell line completely free of EGFR^vIII^ proved impossible to stabilize, in contrast to DK-MG^low^ line composed in vast majority of cells negative for EGFR^vIII^ and only small portion of cells EGFR^vIII^-positive (Figure 2, Sup.Figure 4). The exact number of cells expressing mutant receptor in this cell DK-MG line was around 5% according to FISH analysis and 1% on the protein level (immunofluorescent studies; Figure 2B, 2F and 2G, respectively). Two potential causes underlie such discrepancy - either the methods employed are not sufficiently sensitive to detect changes on the protein level resulting from the small number of amplicons (below 10), or expression of EGFR^vIII^ is somehow regulated post-transcriptionally or epigenetically (e.g. miRNA, protein folding or methylation), which has been reported previously [36, 50, 54, 55]. With regards to the amplicon number and protein levels per cell, the number of EGFR^vIII^-positive cells in the DK-MG^high^ line was much higher compared to the DK-MG^low^ line, however no distinctive populations could be observed in FISH and immunofluorescence studies, indicative of high heterogeneity (Figure 2B and 2F, respectively). Of particular importance is the fact that *EGFR^vIII^* mRNA levels as well as amplicon number did not change over prolonged culturing periods (Sup.Figure 6), even though loss of EGFR^vIII^ amplicons during *in vitro* adaptation process of primary tumor cells has been observed by us (Figure 1C) and consistently reported in the literature [26, 27]. Stability of the DK-MG line, also confirmed by Struve and colleagues [55], is of particular importance in scientific research and drug development, where model's stability is a basic requirement.

Controversial is the role of EGFR^vIII^ in the processes such as populational growth, cell proliferation and viability, with contradicting results reported [25, 40, 44, 55, 56]. However, only a small subset of publications recognizes that populational growth depends on the proliferation rate as well as cell viability, which have opposite effects. We have indicated that BrdU incorporation, a measure of DNA replication essential to the proliferation process, of both DK-MG lines is very similar when not stressed by nutrient deprivation (FBS supplementation) (Figure 3D-3F). Interestingly, proliferation rate of the DK-MG^high^ line in serum free media is trending to be higher than that of DK-MG^low^ line, although statistical significance has not been reached. Those results find confirmation in the molecular studies, which show similar levels of PI3K/Akt signaling pathway activation in both DK-MG cell lines (Figure 4A-4D), pathway that is associated with the proliferation and survival processes, a result identical to the one observed by Struve and colleagues [55, 57, 58]. It should be noted that PI3K/Akt signaling pathway is often referred to as the main signal transducer of the EGFR^vIII^ signaling, which is contradictory to our data [6, 22, 41, 59]. A potential reason of this discrepancy is the hemizygous status of PTEN, the main negative regulator of the pathway, which can upregulate pathway activity in both DK-MG lines irrelevant of EGFR^vIII^ (Figure 2A).

In contrast to the effect of EGFR^vIII^ on proliferation, viability of both DK-MG cell lines differed significantly under all conditions tested (Figure 5A). Cleavage of PARP as well as readout from the synthetic Caspase activity reporter indicate that DK-MG^low^ line cells are more prone to undergo apoptosis (Figure 5B, 5C). This observation is supported by elevated activation of the NFκB pathway in the DK-MG^high^ line, which is strongly associated with pro-survival activity (Figure 6A and 6B) [34, 60]. Interestingly, differences between lines have been observed despite negative regulator of the pathway, *NFKBIA* gene, being heterozygous in both DK-MG cell lines (Figure 2A). This suggests how strong activation of the NFκB pathway by EGFR^vIII^ is. Importantly, suppression of the pathway activity by small molecule inhibitors CID2858522 and ACHP has affected only cells in the DK-MG^high^ line, which confirms a correlation between EGFR^vIII^ expression and NFκB pathway activity, described previously in the literature (Figure 6C) [41, 42, 61]. In contrast to previous reports, EGFRvIII induced NFκB signaling does not result in elevated transcription of a pro-survival factor, *Bcl-XL* in DK-MG cells, as concluded from lack of effect of CID2858522 and ACHP on *Bcl-XL* mRNA levels (Figure 6D) [41, 62]. This is despite the elevated *Bcl-XL* transcript levels in the DK-MG^high^ line (Figure 6D), which might be a result of the activity of the JAK/STAT signaling pathway, reported previously but not investigated by us [44].

Taken together, combination of higher survivability of the DK-MG^high^ line with comparable proliferation rates results in much higher populational growth of cell line enriched in EGFR^vIII^ expression (Figure 3B). Fact that EGFR specific Tyrosine kinase inhibitor, erlotinib, can suppress this growth advantage indicates the central role of EGFR^vIII^. It has to be noted that different growth rate of DK-MG^high^ and DK-MG^low^ lines contradicts recent report from Kriegs lab, who isolated similar subpopulations of DK-MG cells by FACS [55]. Observed differences might be a result of technical differences in cell culture or methodologies applied.

Another important conclusion from our data is the sole fact of establishment of the DK-MG^low^ line implies that even small number of cells expressing EGFR^vIII^ is required for populational survival and growth. Similar conclusions could be drawn from reports of Nathanson et al. as well as Struve et al., who isolated subpopulations of cancer cells with smaller number of EGFR^vIII^-positive cells, however never truly depleted of it, albeit using different methodologies [25, 55]. Studies on the naïve tumor tissue confirm the hypothesis, that small subset of cells positive for EGFR^vIII^ expression is required [14, 15, 25, 63, 64]. A plausible interpretation is that paracrine signaling initiated by EGFR^vIII^-positive cells is required for survival of the cancer associated cells as well as cancer cells negative for EGFR^vIII^ expression. A number of reports indicated paracrine secretion as paramount to glioblastoma growth, with ligands such as TGFα, IL-6, ΙL-8, HGF and heparin binding EGF playing central role, further supporting the hypothesis [16, 26, 42, 65-67]. Obviously, such result does not exclude the possibility that EGFR^vIII^ acts in a cell intrinsic manner, as depicted by previous report on the effect of shRNA mediated silencing of EGFR^vIII^ resulting in cell death [68]. This implies that a combination of cell-intrinsic and -extrinsic effects is relevant and so, heterogenous populations of cells are more appropriate models to study biology of EGFR^vIII^.

Moreover, we have observed strong correlation between EGFR^vIII^ expression level and invasiveness of cell line *in vitro* as well as *in vivo* (Figure 3A and Table 1, respectively). Such correlation is in line with previous reports on the matter [7, 20, 28, 69, 70]. It should be noted, that we cannot conclude whether elevated invasiveness is a cell intrinsic or cell extrinsic effect of EGFR^vIII^ signaling, with both aspects reported in the literature.

Besides functioning as an attractant (Figure 3A), we have observed that EGF positively influenced populational growth of both cell lines (Figure 3B). This result is contradictory to previous reports indicating cells positive for EGFR^vIII^ to undergo apoptosis in response to EGF stimulation [29, 40]. Stimulation of EGFR^vIII^-positive glioblastoma derived neurospheres with EGF produced variable results, whilst U87-MG cells expressing EGFR^vIII^ (same model as in the literature report) did not respond to the stimulus. The contradictions between results within the same model indicate how sensitive model establishment process is, with minor aspects dramatically influencing the outcome.

Finally, we propose that combination of DK-MG cell lines is a suitable model to study the biology of EGFR^vIII^ as well as develop drugs targeting it. With different number of cells expressing EGFR^vIII^ in both lines, drugs targeting the mutant receptor should affect each line to a different extent. At the same time, both lines proved to be highly stable, with no amplicon loss over time (Sup.Figure 6), in contrast to primary cell cultures (Figure 1C). Neurospheres, an alternative model, produced varying results (Figure 1B and Sup.Figure 1) in contrast to reproducible results from DK-MG lines. Finally, DK-MG lines proved highly heterogenous (Sup.Figure 4 and 6) with extrachromosomal EGFR^vIII^ amplicons (Sup.Figure 5), in contrast stable cell lines (Sup.Figure 2) and reminiscent of glioblastoma tissue [14, 15, 25, 63]. Therefore, we propose the use of DK-MG cell lines as an attractive tool for early stage drug development stages, that require more high-throughput approach, while utilizing the patient-derived neurosphere cultures at the later stages of drug validation, giving good measure of drug safety and effectiveness in different genetic makeups. Similarly, basic research can be conducted on DK-MG lines with validation of key concepts performed on the more costly patient derived cells.

## MATERIALS AND METHODS

### Cell culture

DK-MG cells were obtained from DSMZ (Cat. no. ACC-277) and cultured in RPMI 1640 (GIBCO, Life Technologies, Cat. no. 11875-093) medium supplemented with 1% Penicillin/Streptomycin (Life Technologies, Cat. no. 15140-122), 0.2% Gentamycin (Biowest, Cat. no. L0011-100) and 10% FBS (PAA, Cat. no. A15-104) under standard cell culture conditions (5% CO_2_, 16% O_2_, 37°C). Clonal selection was performed by serial dilution in a 96-well plate format.

U87-MG cells were obtained from ATCC (Cat. no. HTB-14™) and cultured in MEM (GIBCO, Life Technologies, Cat. no. 11095-080) medium supplemented with 1% Penicillin/Streptomycin (Life Technologies, Cat. no. 15140-122), 0.2% Gentamycin (Biowest, Cat. no. L0011-100) and 10% FBS (PAA, Cat. no. A15-104) under standard cell culture conditions (5% CO_2_, 16% O_2_, 37°C).

NCI-H460 cells were obtained from ATCC (Cat. no. HTB-177™) and cultured in RPMI 1640 (GIBCO, Life Technologies, Cat. no. 11875-093) medium supplemented with 1% Penicillin/Streptomycin (Life Technologies, Cat. no. 15140-122), 0.2% Gentamycin (Biowest, Cat. no. L0011-100) and 10% FBS (PAA, Cat. no. A15-104) under standard cell culture conditions (5% CO_2_, 16% O_2_, 37°C).

### Construction of pLV1-puro-DEST vector

The pIRESpuro plasmid (Clontech Laboratories, Cat. no. 6031-1) was digested with EcoRI and BamHI restriction enzymes (both New England Biolabs, Cat. no. R0101 and R0136, respectively), blunt-ended with T4 DNA polymerase (New England Biolabs, Cat. no. M0203) and ligated with Gateway Rf. A reading frame cassette. Afterwards, the CMV-GW-IRES-puro fragment was PCR-amplified, digested with HpaI/SnaBI (both New England Biolabs, Cat. no. R0105 and R0130, respectively) and ligated with pLOC-RFP vector (Thermo Scientific, Cat. no. OHS5922) previously digested with Bsu36I/SnaBI enzymes (both New England Biolabs, Cat. no. R0524 and R0130, respectively). Electrophoretic analysis and DNA sequencing were performed to verify the resulting recombinant vectors.

### Preparation of EGFR^vIII^ inserts

Total RNA was isolated from DK-MG cell line (DSMZ, Cat. no. ACC-277) using an AllPrep DNA/RNA Mini Kit (Qiagen, Cat. no. 80204) according to the manufacturer's protocol. cDNA synthesis was conducted using M-MuLV reverse transcriptase (New England Biolabs, Cat. no. M0253) from 500 ng of total RNA. Sequence encoding *EGFR^vIII^* was amplified by PCR using primers 5′ - GGGGACAAGTTTGTACAA AAAAGCAGCGTATGCG ACCCTCCGGGACGGCC - 3′ and 5′ - GGGGACCACTTTGTACA AGAAAGCT GGGTTGCTCCAATAAATTCACTGC - 3′. Subsequently, PCR products were cloned into a shuttle vector pDONR/Zeo^TM^ (Life Technologies, Cat. no. 12535-035) by BP Clonase II Enzyme Mix (Life Technologies, Cat. no. 11789-100) according to the manufacturer's protocol. Resulting recombinant plasmids were then cloned into pLV1-puro-DEST lentiviral vectors by LR Clonase II Enzyme Mix (Life Technologies, Cat. no. 11791-100) following the manufacturer's recommendations. Alternatively, EGFR^vIII^ was amplified with 5′ - GACTG CTAGCCGCCACCAT GCGACCCTCCGGGACGGCC - 3′ and 5′- GACTTTCGAATCATGCTCCAATAAATTCACTGC-3′ and PCR products were cloned into a shuttle vector pIRESneo3 (Clontech Laboratories, Cat. no. 631621) sites by classical cloning with the use of NheI/BstBI restriction enzymes (both New England Biolabs, Cat. no. R0131 and R0519, respectively), Shrimp Alkaline Phosphatase (New England Biolabs, Cat. no. M0371) and T4 DNA Ligase (New England Biolabs, Cat. no. M0202).

### Lentivirus production

Lentivirus carrying *EGFR^vIII^* sequence was prepared using LENTI-Smart kit (InvivoGen, Cat. no. Its-str) following the manufacturer's recommendations in HEK293T cell line (ATCC, Cat. no. CRL-1573™). HEK293T cells were seeded at a density of 7×10^6^ cells/10 cm dish and grown in DMEM High glucose (Biowest, Cat. no L0102) supplemented with 10% FBS (PAA, Cat. no. A15-104) and incubated at 37°C, 5% CO_2_. Twenty four hours after the initial plating, transfection medium containing LENTI-Smart and lentiviral transfer plasmids was added. The cell culture medium was changed the following day and collected for the next two days. Cellular debris was removed from the supernatants by filtration through a 0.45 μm filter. Lentiviral molecules were concentrated with 100 kDa cut-off Amicon Ultra centrifugal filter (Merck Millipore, Cat. no. UFC910024). HT-1080 (ATCC, Cat. no. CCL-121™) cell line was used to titter lentiviral vectors based on puromycin resistance.

### Transduction of NCI-H460 and clonal selection

NCI-H460 cells were seeded at 1×10^5^ cells per well in a 6-well plate, allowed to adhere for 24h and transduced with equal amounts of lentivirus encoding EGFR^vIII^. Cells were selected using puromycin (5 μg/mL; InvivoGen, Cat. no. ant-pr-1) supplemented complete growth media for 5 days. Puromycin was used throughout cell culture for maintenance purposes (1 μg/mL).

### Transfection of U87-MG and clonal selection

Transient transfection was performed with Lipofectamine^®^ LTX Reagent (Life Technologies, Cat. no. 15338-100) following the manufacturer's protocol. Briefly, on day prior transfection the U87-MG cells were plated at 0.5×10^6^ cells/well on a 6-well plate in complete growth medium. At approximately 70% confluency, medium was changed for fresh complete growth medium and cells were transfected. 12 μL of Lipofectamine LTX Reagent per well was diluted in 150 μL Opti-MEM^®^ Medium (GIBCO, Life Technologies, Cat. no. 31985-070) and 2.5 μg of each plasmid DNA (pIRESneo3, pIRESneo3/gw EmGFP and pIRESneo3-EGFR^vIII^) per well was diluted in another 150 μL Opti-MEM^®^ Medium. After that, 2.5 μL of Plus^®^ Reagent was added into Opti-MEM^®^ Medium with diluted DNA. Diluted DNA with Plus^®^ Reagent and diluted Lipofectamine^®^ LTX Reagent were mixed at ratio 1:1, incubated for 10 min in RT and then 250 μL of DNA-reagent complex was added into each well and cells were incubated at 37°C for 24h post-transfection (before assaying for transgene expression and selection).

Transfected cells were positively selected for 7 days in G418 (0.4 mg/mL; InvivoGen, Cat. Code ant-gn-1). Approximately 10 days after transfection, positively selected colonies were picked, transferred into separate wells of 24 well plates for further propagation.

### Invasion assay

Invasion assay was performed using BioCoat Matrigel Invasion Chambers in 24-well plate format (Corning, Cat. no. 354480), according to manufacturer instructions. Briefly, DK-MG cells from both lines were seeded at 1×10^4^ per transwell in medium supplemented with 10% FBS (PAA, Cat. no. A15-104) on both sides. Medium was changed to serum free media after 24h and EGF at 20 ng/mL (Sigma, Cat. no. E9644) was added to the medium in the lower chamber. After three days of incubation, transwells were prepared into slides with Coomassie Blue used for cell staining. Images from seven random fields of view were obtained from each transwell and the average number of invasive cells was determined.

### Viability assay

Cells seeded at 2×10^5^/well in 6-well plates were left to adhere overnight and incubated for 3 days in serum free medium on its own or supplemented with 10% FBS (PAA, Cat. no. A15-104) or 20 ng/mL EGF mL (Sigma, Cat. no. E9644). At this point, cells were scraped off without any medium change and viability assay was carried out using AccuChip^4x^ kit on ADAM (Digital Bio) according to manufacturer guidelines.

### Tumor samples

Tissue samples were obtained from patients diagnosed with glioblastoma undergoing tumor resection at the Department of Neurosurgery, Hospital of Saint Raphael in Krakow or at the Clinical Department of Neurosurgery, The Voivodal Specialistic Hospital in Olsztyn. All samples were collected using the protocol approved by the Bioethical Committee of the Medical University of Lodz (Approval No RNN/9/10/KE). Written informed consent was obtained from all patients and their data were processed and stored according to the principles expressed in the Declaration of Helsinki. The patients were diagnosed according to the World Health Organization Criteria for Brain Tumor Classification (2007).

Isolation of cells from fresh glioblastoma specimens started within 3 hours after neurosurgical operation. Neurosurgical specimens were shipped in 1x Hank's Balanced Salt Solution (Biowest, Cat. no. L0607).

### Establishment and growth of GB cells under the classical culture conditions

Fresh glioblastoma samples were washed twice with 1x Hank's BSS (Biowest, Cat. no. L0607), centrifuged for 90 s at 80 x g each time, cut into < 1 mm^3^ fragments, washed again with 1x Hanks’ BSS and digested with collagenase type IV and dispase (both 200 U/mL; GIBCO, Life Technologies, Cat. no. 17104-019, StemCell Technologies, Cat. no. 07923, respectively) on shaker for 30 min at 37°C. Digested tissue was filtered using a 70 μm cell strainer (BD Biosciences, Cat. no. 352350). Filtered cells were washed twice with 1x Hank's BSS with 90 s centrifugation at 80 × g and seeded onto 6-well plates at 2.5-5×10^5^ cells/well. Cells were cultured in Neurobasal Medium (GIBCO, Life Technologies, Cat. no. 21103-049) with B27 supplement (20 μl/mL; GIBCO, Life Technologies; Cat. no. 17504-044), Glutamax (10 μl/mL; GIBCO, Life Technologies; Cat. no. 35050-061), fibroblast growth factor-2 (20 ng/mL; Sigma, Cat. no. F0291), NEAA (GIBCO, Life Technologies, Cat. no. 11140-050) and heparin (2 μg/mL; StemCell Technologies, Cat. no. 07980). Growth factors and heparin were added twice a week. To assess effects of EGF or erlotinib, neurospheres were moved to new plates and cultured in AGM™ Astrocyte Growth Medium (Lonza, Cat. no. CC-3187) with AGM BulletKit (Lonza, Cat. no. CC-3186) for 24h to allow adhesion and either EGF (20 ng/mL), erlotinib (10 mM) or DMSO, where appropriate, were added. Neurospheres were imaged using BioStation CT, as described for stable cell lines. To establish stable cell lines, spheres were split by mechanical dissociation and transferred to new dish when they reached the size of 200-500 μm. Cells were cultured under normal culture conditions in AGM™ Astrocyte Growth Medium with AGM BulletKit.

### MLPA

Multiplex Ligation-dependent Probe Amplification (MLPA) was carried out using P105-D1 Glioblastoma-2 probemix (MRC-Holland, Cat. no. D1-0413) and SALSA MLPA EK1 kit - FAM (MRC-Holland, Cat. no. EK1-FAM), as described in [27].

### Inhibitors used

DK-MG cells were seeded in 6-well plates at 2×10^5^ cells per well for western blot analysis or at 4×10^4^ cells per well for real-time observation, incubated overnight to allow for adhesion and serum starved for subsequent 24h. Serum free medium containing appropriate inhibitors was applied onto cells and incubated for indicated amount of time, prior to stimulation with 20ng/mL EGF (Sigma-Aldrich) or 5ng/mL TNFa (Cell Signaling Technology) for 20 or 30min, as indicated, prior to lysis for western blotting or left for real-time observation. Following concentrations of inhibitors were used: DMSO (Sigma, Cat. No.D2438, solvent control), 10 μM Erlotinib (Molecula; Cat. No. 89983631), 2.5 μM ACHP (Tocris Bioscience, Cat. No. 4547), 1 μM CID2858522 (Tocris Bioscience, Cat. No. 4246).

### Western blotting

Cells were lysed in cell lysis buffer (50 mM Tris-HCl pH 7.5, 1 mM EDTA, 1 mM EGTA, 1 mM Sodium Orthovanadate, 10 μM β-glycerophosphate, 5 μM Sodium Pyrophosphate and 0.5% Triton X-100) freshly supplemented with Protease Inhibitor Cocktail (Sigma, Cat. no. P8340) at 4°C. Proteins were purified by centrifugation for 5 min at 7,000 x g, separated on 8% SDS-PAGE and transferred onto the PVDF membrane (Immobilon - P, Merck Millipore, Cat. no. IPVH00010). Membrane was blocked with 5% PhosphoBlocker Blocking Reagent (Cell Biolabs, Cat. no. AKR-104) and probed with antibodies listed in Sup.Table.2. Bands were visualized using Amersham ECL Prime Western Blotting Detection Reagent (GE Healthcare, Cat. no. RPN2232) on ChemiDoc XRS (Bio-Rad).

### BrdU incorporation, immunofluorescence and image analysis

5-bromo-2′-deoxyuridine incorporation assay as well as immunofluorescent analysis was performed as described previously [27]. Briefly, cells were fixed in 4% PFA/PBS, permeabilized with 0.1% Triton-×100/PBS and blocked with 2% donkey serum (Sigma, Cat. no. D9663) and incubated with appropriate antibodies (Sup.Table.2). For EGFR^WT^ degradation experiment, cells were stimulated with EGF (20 ng/mL;Sigma, Cat. no. E9644) for 1h prior to fixation. Alexa Fluor 568 Phalloidin (Life Technologies, Cat. no. A12380) was incubated at 1:50 together with secondary antibodies, according to manufacturer guidelines. Following fixation of slides with Prolong Gold with DAPI (Life Technologies, Cat. no. P36935), slides were imaged on Nikon Eclipse Ci (Nikon, Tokyo, Japan) fluorescent microscope, images were captured using NIS - Elements F (Nikon, Tokyo, Japan) Software and processed using ImageJ software [71]. In case of a synthetic Caspase 3/7 activity reporter - CellEvent Caspase-3/7 Green (Life Technologies, Cat. no. R37111) - live cells were incubated with the solution for 1h according to manufacturer guidelines. Cells were fixed and imaged as described above. Obtained images were analyzed using CellProfiler freeware analysis software [72]. At least 100 cells were used per single analysis.

### Quantitative real-time PCR

Total RNA was isolated as described for NGS. Concentration of nucleic acids was measured spectrophotometrically (NanoPhotometer, Implen) and 250 ng of total RNA was used for reverse transcribed using a QuantiTect Rev. Transcription Kit (Qiagen, Cat. no. 205311) according to the manufacturer's protocol. 50 ng of prepared cDNA was used in qRT-PCR reaction as a template along with 200 nM of each primer, 6 μL of SYBR Select Master Mix (2X) (Life Technologies, Cat. no. 4472908) and total volume of 12 μL on StepOne-Plus Real-Time PCR System (Life Technologies). Sup.Table.3 summarizes primer sequences used. The cycling conditions were as follows: 2 min at 50°C, 10 min at 95°C, followed by 40 cycles of: 15 s at 95°C, 30 s at 60°C and 30 s at 72°C. To confirm the specificity of the amplification signal, the gene dissociation curve was considered in each case, using LinRegPCR software. Normalized relative expression level was calculated utilizing the method described previously by Pfaffl and colleagues [73], based on each sample's average Ct value and each gene's average PCR efficiency. The pool for qRT-PCR containing EGFR^vIII^ that does not occur in normal tissue, mRNA isolated from 15 cancer tissues positive for mutant receptor was pooled and diluted 50 times.

### Real time cell observation

DK-MG cells were seeded at 4×10^4^ cells/well in 6-well plates, left overnight to adhere in full medium, serum starved for 24h, pre-treated with indicated inhibitors for 1h and incubated in serum free medium supplemented with 20 ng/mL of EGF (Sigma, Cat. no. E9644) or 10% FBS (PAA, Cat. no. A15-104) as indicated. At this time plate was placed in BioStation CT (Nikon) and incubated for 5 days under normal culture conditions (16% O_2_, 5% CO_2_). Every 6h five images per well were taken at 10 x magnification, with first set of images taken within 3h following loading into the machine. Cell counts were performed using CL-Quant Software v3.10 (DRVision Technologies).

### Mice inoculation and measurement

Crl:SHO-PrkdcscidHrhr mice were delivered by Animalab (Poznan, Poland) and all studies involving mice were conducted in accordance with federal and institutional guidelines. Xenograft tumors were generated as described in [74]. Briefly, DK-MG cells cultured under standard conditions were collected by centrifugation, supernatant was discarded and cell clumps eliminated. 2×10^6^ cells were mixed with equal volume of Matrigel (BD Biosciences, Cat. no. 356234) and injected subcutaneously on the right/left flank of 5-6 week old mice. The two longest perpendicular axes in the x/y plane of each xenograft tumor were measured 3 times a week with an electronic caliper and tumor volume was calculated according to formula: 0.5xy^2^ [75]. After 6 weeks, mice were sacrificed.

### Reproducibility and statistical analysis

All experiments were conducted in at least three replicates and representative images/graphs are shown. Relevant statistical analysis method is indicated in the figure legend. Analysis was performed in GraphPad Prism 5 (Graphpad Software).

## SUPPLEMENTARY MATERIAL FIGURES AND TABLES




