# Supplementary material for: Cell line with endogenous EGFRvIII expression is a suitable model for research and drug development purposes
Source: Oncotarget. 2016 Mar 19;7(22):31907–25. doi: 10.18632/oncotarget.8201 (PMC5077985; doi:10.18632/oncotarget.8201)
Supplement: Supplementary file 1 [file oncotarget-07-31907-s001.pdf]

Cell line with endogenous $EGFR^{vIII}$ expression is a suitable model for research and drug development purposes

Supplemental Materials

Materials and methods

Next Generation Sequencing

The high-quality whole genomic DNA for sequencing was isolated using AllPrep DNA/RNA Kit (Qiagen, Valencia, CA, USA). The libraries were prepared using Ion AmpliSeq™ Comprehensive Cancer Panel (Life Technologies, Carlsbad, CA, USA). The quality and concentration of libraries was estimated on Agilent BioAnalyzer with a High Sensitivity DNA Chip (Agilent, Santa Clara, CA, USA).

The emulsion PCR and Ion Sphere Particles enrichment was carried out according to the original Life Technology protocols on respectively Ion OneTouch2 Instrument and Ion OneTouch ES instrument (Life Technologies, Carlsbad, CA, USA). Final sequencing process was performed on Personal Genome Machine (PGM) using Ion 318™ Chip (Life Technologies, Carlsbad, CA, USA).

Obtained data, generated by the Torrent Suite Software version 4.0.2 with a Variant Caller version 4.0, were aligned against the human genome (hg19) reference sequence and visualised by Integrative Genomics Viewer (IGV). For analysis, following parameter thresholds were used: Minimum quality>10, Minimum coverage> 20, Minimum coverage on either strand>3, Maximum strand bias< 0.95 (for SNP and Hotspots), and <0.85 (for Indels), Minimum relative read quality >8.5, Maximum common signal shift <0.25, Maximum reference/variant signal shift <0.2, Maximum homopolymer length <8.

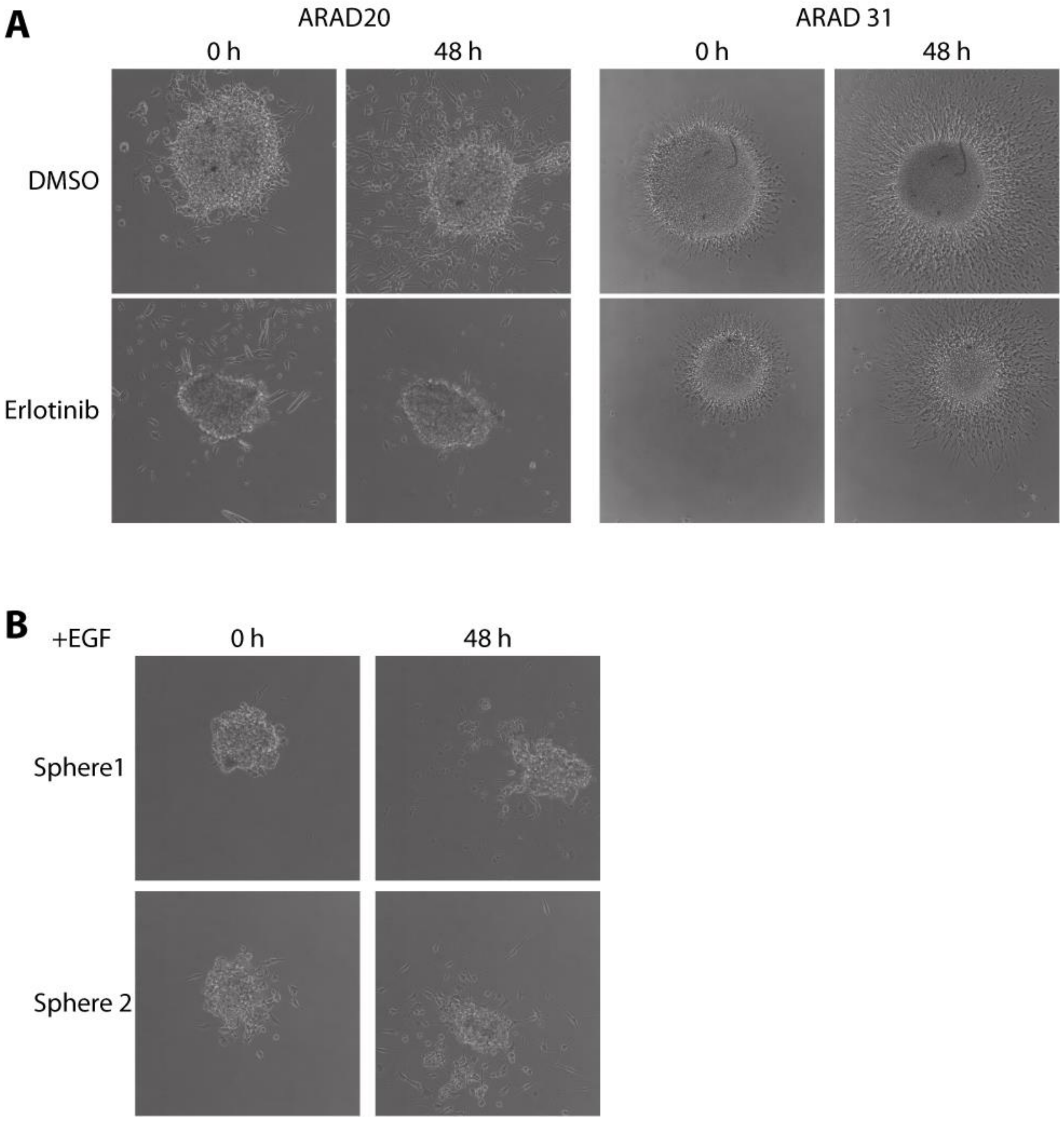

Supplemental Figure 1. Neurospheres from glioblastoma primary cells display high variability in response to erlotinib or EGF treatment. (A) Representative images of adherent neurospheres from glioblastomas positive for $EGFR^{vIII}$ expression treated with DMSO or 10µM erlotinib. Live cells migrating from neurosphere indicate no effect of treatment. (B) Adherent neurospheres from ARAD20, positive for $EGFR^{vIII}$ expression, react differently to EGF treatment (20ng/mL), as indicated by the state of cells surrounding the neurosphere (dead in sphere 1 and live in sphere 2). Images captured by Biostation CT using 10x magnification objective.

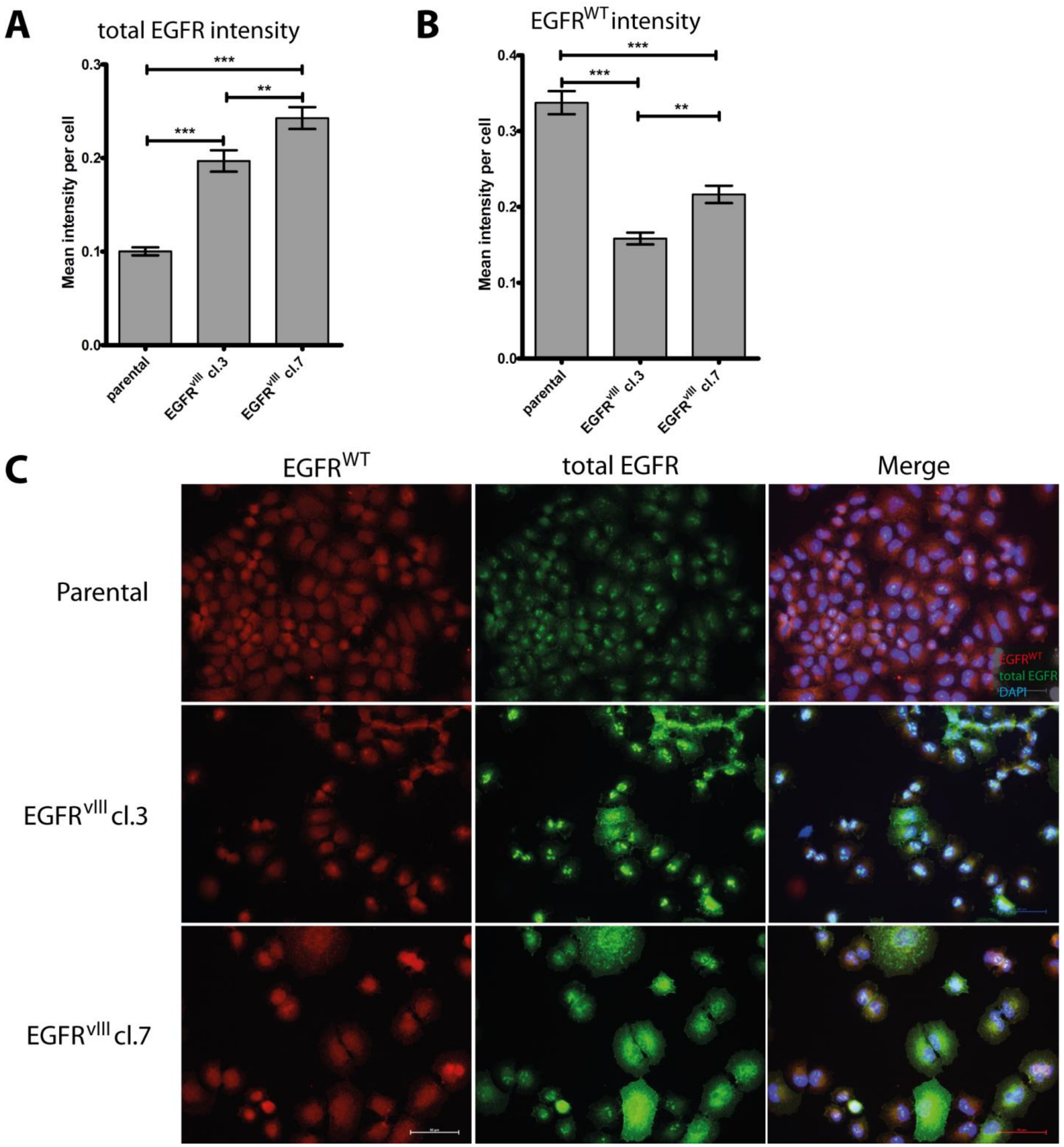

Supplemental Figure 2. Comparison of parental NCI-H460 cell line to two clones transduced with *EGFR^{vIII}* carrying virus. (A-C) Cells were stained with antibodies recognizing the epitope specific to EGFR^{WT} and epitope common to wild-type and mutant receptors. (A) Quantification of mean intensity of staining obtained by total EGFR staining. (B) Quantification of mean intensity of staining obtained by EGFR^{WT} staining. (C) Representative images, depicting staining for EGFR^{WT} (left panel), total EGFR (middle panel) and their merge with DAPI (right panel). Error bars indicate SEM. Statistical analysis performed with one-way ANOVA with post-analysis Bonferroni's multiple comparison test. ***, $p<0.05$; **, $p<0.01$; *, $p<0.005$; ns, not significant. 20x objective used.

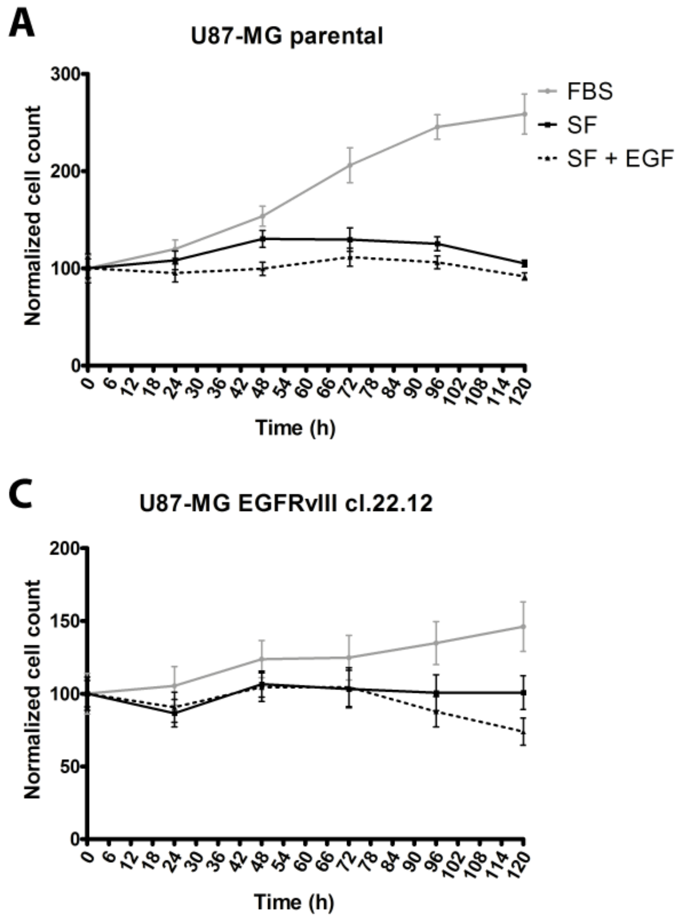

Supplemental Figure 3. Effects of exogenous EGFR^{vIII} on U87-MG cell line. (A-D) Populational growth rate of monoclonal U87-MG cell lines without *EGFR^{vIII}* inserted (A) or three independent stably expressing the mutant receptor clones (B-D) was assessed under different culture conditions, in serum free media (SF), supplemented with EGF at 20ng/mL (SF + EGF) or with 10% FBS supplementation (FBS). Error bars indicate SEM.

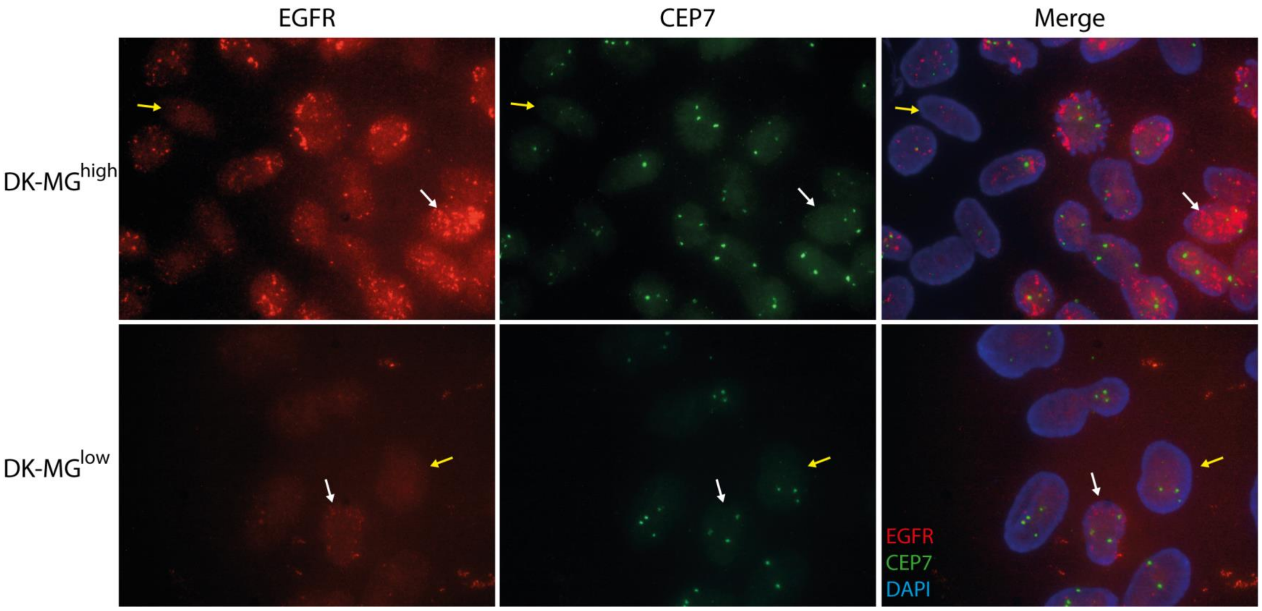

Supplemental Figure 4. The number of cells positive for *EGFR^{vIII}* amplicons varies between two DK-MG lines. FISH analysis of DK-MG^{high} (upper panel) and DK-MG^{low} (lower panel) lines indicates drastic difference in the ratio of cells positive for EGFR^{vIII} amplicons (white arrows) and negative cells (yellow arrows). Images obtained using 60x magnification objective.

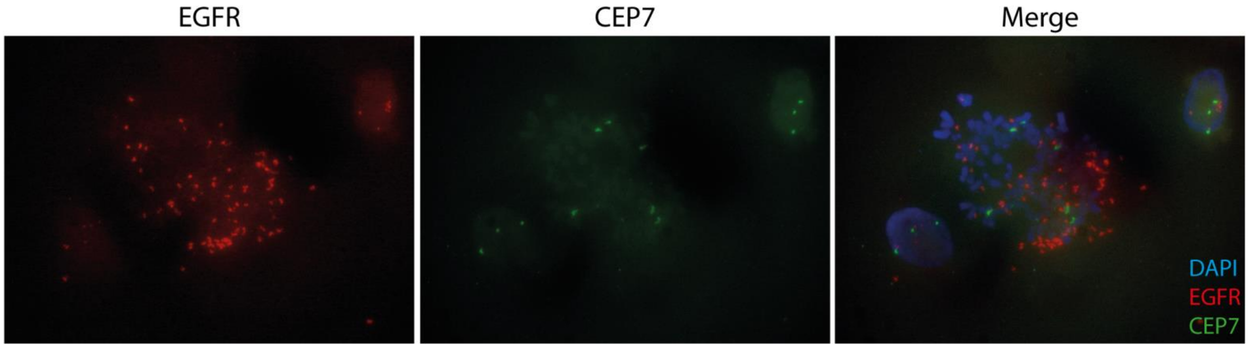

Supplemental Figure 5. DK-MG cells have extrachromosomal EGFR amplicons. FISH analysis of DK-MG cell undergoing mitosis. Extrachromosomal amplicons are visible. Images obtained using 100x magnification objective.

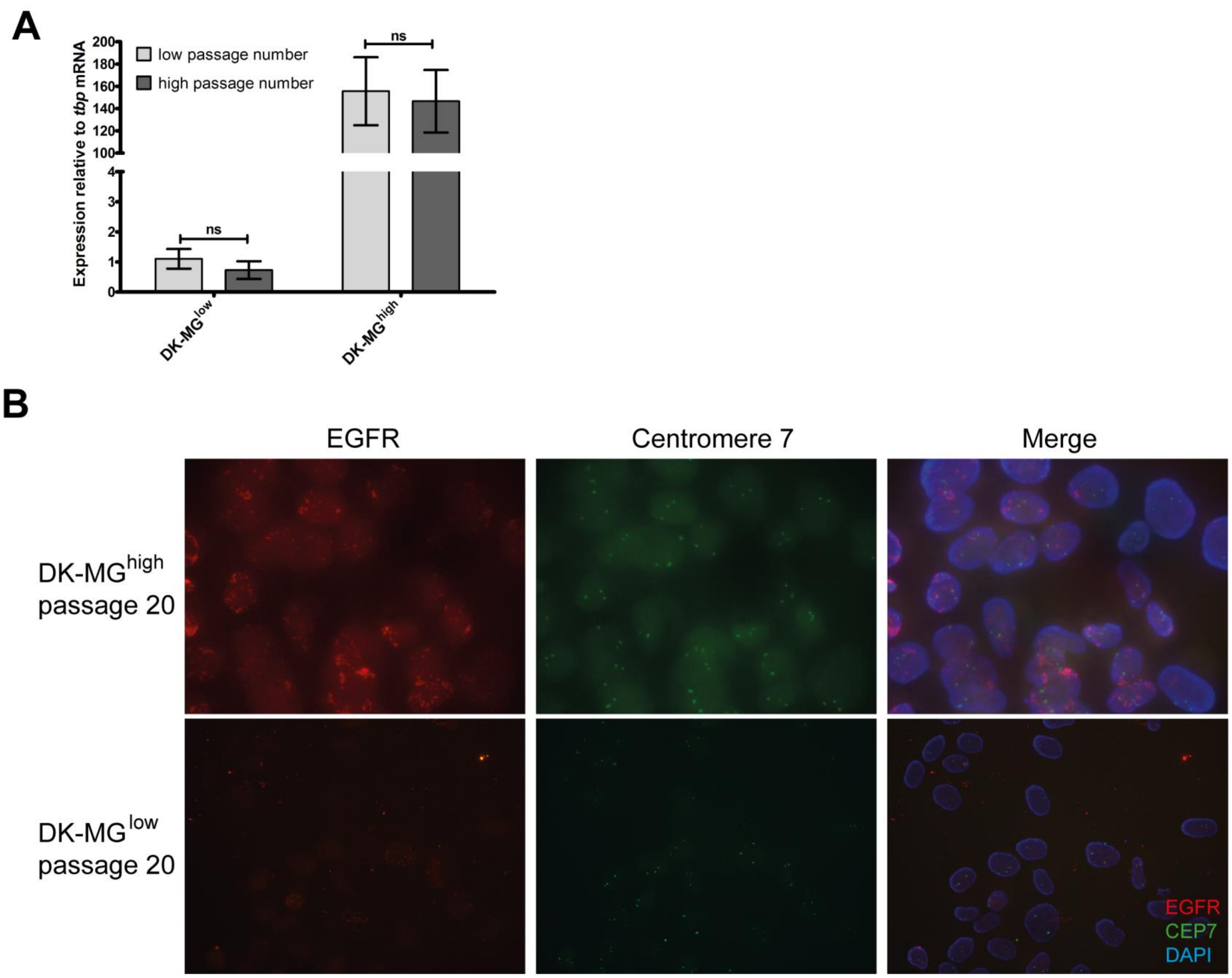

Supplemental Figure 6. *EGFR^{vIII}* mRNA levels and amplicon numbers in DK-MG cell lines do not change across passages. (A) Total mRNA was isolated from both DK-MG cell lines in low or high passage number and analyzed by qRT-PCR for *EGFR^{vIII}* expression. Passage between 2 and 5 – described as low passage number, passage between 18 and 22 – described as high passage number. (B) DK-MG^{high} and DK-MG^{low} cells cultured for 15 passages following clonal selection were analyzed by FISH. No significant change over time in amplicon number and composition of populations in regards to the number of cells positive for EGFR amplicons, has been noticed for both cell lines. Images obtained using 60x magnification objective.

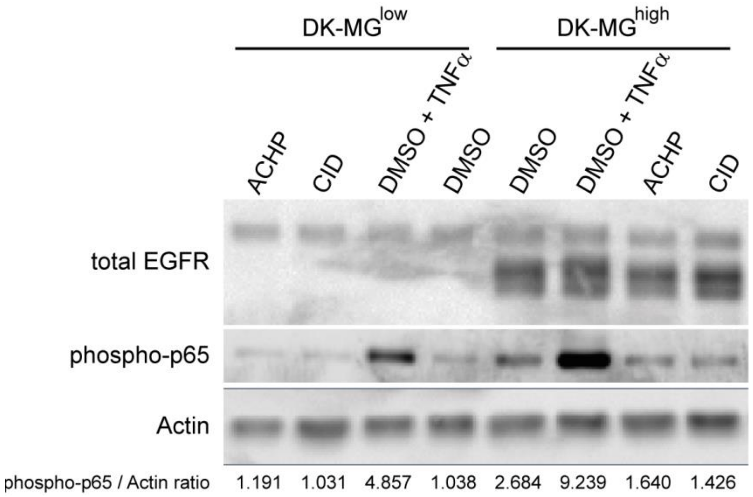

Supplemental Figure 7. Phosphorylation of p65 is mildly suppressed by NFκB pathway inhibitors and induced by TNFα treatment. DK-MG^{high} and DK-MG^{low} were treated with DMSO, ACHP or CID2858522 inhibitors for 3 days prior to stimulation with TNFα for 20 min, as indicated, prior to lysis and analysis via western blotting. Numbers beneath the blots indicate the ratio of band density of the phosphorylated p65 over actin.

Sup. Table 1. Next Generation Sequencing of DK-MG cell lines. Table represents Ion Torrent PGM sequencing data for DK-MG^{high} and DK-MG^{low} cell lines. Mutations reported to be different between lines (marked in grey) were analysed on raw data using Ion Reporter 4.4 Software and Integrative Genomics Viewer for confirmation. All of them proved to be questionable, due to low coverage, bad read quality or software's misinterpretation. Selected few were confirmed by Sanger sequencing to be false positive results.

Supplemental Table 2. List of antibodies used in immunofluorescence staining and western blot analysis.

ANTIBODIES USED IN WESTERN BLOT			
**Antibody**	**Host**	**Manufacturer**	**Dilution**
anti-EGFR (1005)	rabbit	Santa Cruz Biotechnology, Inc., sc-03; Dallas, TX, USA	1 : 200
anti- PARP	rabbit	Cell Signaling Technology, Inc., 9542; Danvers, MA, USA	1:1000
anti-Actin, clone C4	mouse	Merck Millipore, MAB1501; Billerica, MA, USA	1 : 8000
anti – rabbit IgG-HRP	goat	Santa Cruz Biotechnology, Inc., sc-2004; Dallas, TX, USA	1 : 4000
anti – mouse IgG-HRP	goat	Santa Cruz Biotechnology, Inc., sc-2005; Dallas, TX, USA	1 : 4000
anti – Akt	rabbit	Cell Signaling Technology, Inc. 9272; Danvers, MA, USA	1 : 500
Anti – phosphorylated S473 Akt (D9E)	rabbit	Cell Signaling Technology, Inc. 4060; Danvers, MA, USA	1 : 1500
Anti – phosphorylated T308 Akt (D25E6)	rabbit	Cell Signaling Technology, Inc. 13038; Danvers, MA, USA	1 : 1000
Anti – phosphorylated p65 (Ser536)	rabbit	Cell Signaling Technology, Inc. 3033; Danvers, MA, USA	1 : 1000
**ANTIBODIES USED FOR IMMUNOFLUORESCENT STAINING**			
**Antibody**	**Host**	**Manufacturer**	**Dilution**
anti-EGFR (528)	mouse	Santa Cruz Biotechnology, Inc., sc-120; Dallas, TX, USA	1 : 50
anti-EGFR (1005)	rabbit	Santa Cruz Biotechnology, Inc., sc-03; Dallas, TX, USA	1 : 250
anti-EGFR (R-1)	mouse	Santa Cruz Biotechnology, Inc., sc-101; Dallas, TX, USA	1 : 50
anti-BrdU	mouse	Sigma-Aldrich, B 8434; St. Louis, MO, USA	1 : 500
anti-mouse Alexa Fluor®594	donkey	Molecular Probes, Invitrogen; Carlsbad, CA, USA	1 : 500
anti-rabbit Alexa Fluor®488	donkey	Molecular Probes, Invitrogen; Carlsbad, CA, USA	1 : 500
Anti – phosphorylated S473 Akt	rabbit	Cell Signaling Technology, Inc. 4060; Danvers, MA, USA	1 : 250

Supplemental Table 3. Primer sequences used for quantitative real-time PCR.

Primer	Sequence
TBP_F	5’ GAGCTGTGATGTGAAGTTTCC 3’
TBP_R	5’ TCTGGGTTTGATCATTCTGTAG 3’
$EGFR^{vIII}$_F	5’ GGCTCTGGAGGAAAAGAAAGGTAATTATGT 3’
$EGFR^{vIII}$_R	5’ ACCAATACCTATTCCGTTACACACT 3’
$EGFR^{WT}$_F	5’ TAGCAGTCTTATCTAACTATGAT 3’
$EGFR^{WT}$_R	5’ CACTGCTGACTATGTCCCGC 3’
Bcl-XL_F	5’ TCCATCTCCGATTCAGTCCCT 3’
Bcl-XL_R	5’ GAGCTGGTGGTTGACTTTCTC 3’
